# Skin manifestations in diabetes—what is new?

**DOI:** 10.3389/fmed.2025.1640144

**Published:** 2025-10-16

**Authors:** Natalia Dorf, Mateusz Maciejczyk

**Affiliations:** ^1^Independent Laboratory of Cosmetology, Medical University of Białystok, Bialystok, Poland; ^2^Department of Hygiene, Epidemiology and Ergonomics, Medical University of Białystok, Bialystok, Poland

**Keywords:** diabetes mellitus, diabetes complications, oxidative stress, skin, diabetic skin

## Abstract

Diabetes is a chronic disease with a continuously increasing prevalence worldwide. Chronic hyperglycaemia results from elevated blood glucose levels due to disturbed insulin secretion and/or action. Diabetes adversely affects the structure and function of micro- and macrovasculature, leading to the failure of various organs and tissues. Diabetes complications affect the kidneys, retina, peripheral nerves, heart, brain, muscle, and skin. Approximately 30% of diabetic patients have cutaneous manifestations, which may be the first sign of metabolic derangement. Skin manifestations strongly associated with diabetes are foot ulcers, diabetic gangrene, diabetic dermopathy, yellow palms and soles, acanthosis nigricans, bullosis diabeticorum, diabetic thick skin, scleredema diabeticorum, and necrobiosis lipoidica. Non-specific symptoms associated with diabetes include acrochordons, rubeosis faciei diabeticorum, eruptive xanthomas, acquired reactive perforating collagenosis, keratosis pilaris, pruritus, vitiligo, granuloma annulare, lichen planus, as well as bacterial and fungal infections. The prompt recognition of skin lesions can initiate early diagnostic testing and timely treatment, minimising long-term complications of diabetes. The use of specialised bioactive dressings in the treatment of diabetic wounds, as well as immunomodulatory and anti-fibrotic therapies in diabetic dermatoses, is a current treatment trend. This review summarises the recent knowledge on the pathogenesis and clinical conditions of cutaneous manifestations related to diabetes mellitus.

## 1 Introduction

Diabetes mellitus (DM) is a group of metabolic disorders characterised by chronic hyperglycaemia. People of any geographic or racial origin can suffer from an elevated blood glucose level, which results from disturbed insulin secretion and/or insulin action (1). DM is a significant global public health problem. Analysis of the past three decades reveals that the prevalence of DM has increased fourfold, especially in developing countries. The global incident cases of DM are estimated to be 463 million (9.3% refers to adults aged 20–79), and by 2045, this number is predicted to increase to 700 million (2). The alarming rate of increase in DM represents the most significant and challenging health problem in the human population of the present world (3).

According to the World Health Organization (WHO), DM can be divided into two main types (1). T1DM can develop at any age but is usually considered a childhood disease. However, new data indicate that up to 42% of T1DM cases occur in patients after 30 years of age, often being initially misdiagnosed as type 2 (3). T1DM is characterised by the autoimmune destruction of the insulin-producing pancreatic *β*-cell islets, which usually leads to absolute insulin insufficiency. Without enough insulin, glucose levels increase in the bloodstream. As a result, patients with DM have persistent hyperglycaemia. T2DM is much more prevalent and is typically associated with adulthood. It is almost always related to insulin resistance in peripheral tissue and impaired insulin secretion due to *β*-cell failure (3, 4). Chronic hyperglycaemia in DM contributes to abnormalities in carbohydrate, lipid, and protein metabolism. These disruptions negatively impact the structure and function of micro- and macrovascular systems, leading to damage, dysfunction, and eventual failure of various organs and tissues. If left untreated, the disease causes several life-threatening medical complications affecting the eyes, kidneys, and nerves (5). Retinopathy, nephropathy, and neuropathy are forms of microvascular complications, while heart attack, hypertension, hyperlipidemia, strokes, as well as coronary and peripheral vascular disease are associated with macrovascular complications. These long-term DM-related complications reduce the quality of life and have a clinically important impact on an increase in the diabetes-associated mortality rate (5, 6). The risk of cardiovascular disease doubles in patients with hyperglycaemia, and about 75% of deaths are due to coronary vascular disease (7).

The complications of DM affect every organ system, including the skin. Approximately 30% of diabetic patients have cutaneous manifestations, which may be the first sign of metabolic derangement (8, 9). Skin manifestations strongly associated with DM are foot ulcers, diabetic gangrene, diabetic dermopathy, yellow palms and soles, acanthosis nigricans, bullosis diabeticorum, diabetic thick skin, scleredema diabeticorum, and necrobiosis lipoidica (8, 10). Non-specific symptoms associated with DM include acrochordons, rubeosis faciei diabeticorum, eruptive xanthomas, acquired reactive perforating collagenosis, keratosis pilaris, pruritus, vitiligo, granuloma annulare, lichen planus, and bacterial and fungal infections (8, 11, 12). Prompt recognition of skin lesions is essential, as it enables early diagnostic testing and timely treatment, minimising long-term complications of DM (10). The pathogenesis of these cutaneous manifestations is multifactorial. The underlying causes are biochemical, vascular, immune, and metabolic changes that occur in the diabetic state (8, 13, 14). The complexity of the mechanisms linking all diabetic complications is crucial for understanding a holistic approach to DM management. The rising costs of healthcare and challenges in effectively treating diagnosed diabetes make it an ideal target for preventive measures to reduce future medical complications (15). This is particularly important in the post-coronavirus disease-19 (post-COVID-19) era, which has increased the incidence of new-onset DM (16). There are some dermatological manifestations of post-COVID-19 syndrome, such as hair loss, subcutaneous nodules, dermatitis, oedema, pigmentation changes, pruritus, or blisters (17). Patients with DM may develop and experience worsening dermatological complications, which may explain the prevalence of diabetes and dermatitis in the post-COVID era (18).

Appropriate care for diabetic skin includes preventing, detecting, and managing skin lesions. Difficult-to-heal wounds and inflammatory skin conditions pose a significant clinical challenge (19). To achieve an overall improvement in skin condition, a comprehensive and holistic approach is necessary, incorporating natural products such as lutein, curcumin, resveratrol, or mangiferin (20). Silver nanoparticles are also a promising agent for the treatment of diabetic wounds and ulcers (19). This review summarises current knowledge on the pathogenesis and clinical conditions of cutaneous manifestations related to DM.

## 2 Pathophysiology of DM

Glucose is the main energy source in organisms. It is derived from the intestinal absorption of food, glycogenolysis (the breakdown of glycogen, which is a stored form of glucose found in the liver), and gluconeogenesis (a metabolic pathway that results in the synthesis of glucose using non-carbohydrate precursors such as lactate, glycerol, and glucogenic amino acids) (21). In response to disturbed glucose homeostasis, two major hormones play a crucial role in the stabilisation of glucose content in the blood—insulin and glucagon (22). Insulin is responsible for controlling the uptake of glucose from the blood into most cells of the body, especially the liver, skeletal muscles, and adipose tissue. The sugar-lowering properties of insulin result from its ability to inhibit the breakdown of glycogen and the gluconeogenesis pathway, as well as its ability to induce glucose transport into fat and muscle cells (23). Therefore, deficits in insulin production contribute to the pathogenesis of both DM1 and DM2 (24). Insulin is a highly effective hormone produced by *β*-cells found in the Langerhans islets of the pancreas. β-cells, in response to high levels of glucose, secrete insulin into the blood, and when glucose levels are low, they decrease the production of insulin. Their neighbouring cells—*α*-cells—function oppositely. α-cells secrete glucagon into the blood in response to lower glucose content and inhibit its secretion when glucose concentration is adequate. Glucagon increases blood glucose by stimulating the gluconeogenesis pathway and glycogenolysis (25). Intrapancreatic hormone interactions result in stable blood glucose levels through precise coordination of glucose production and glucose uptake (23, 24, 26).

Chronic hyperglycaemia plays a major role in the initiation of DM. The kidneys are not able to absorb all circulating glucose, and the excess glucose is excreted out of the body through urine (glycosuria). The osmotic pressure of the urine increases and inhibits the reabsorption of water by the kidneys, leading to increased urine production (polyuria). In this case, diabetics produce a high volume of glucose-containing urine. Water from body cells is used to fill the lost blood volume, resulting in dehydration and increased thirst (polydipsia) (27). As a consequence of persistently high blood glucose levels, many metabolic disorders occur, such as metabolic ketoacidosis (DKA) (28). It is a medical emergency resulting from the destruction of *β*-cells and absolute insulin deficiency, which causes the liver to convert triglycerides from fat into ketone bodies. These ketone bodies enter the circulation mostly as *β*-hydroxybutyrate and acetoacetate, making the blood acidic (29, 30). Excessive production of ketone bodies manifests as nausea, vomiting, abdominal pain, deep breathing known as Kussmaul breathing, and the smell of acetone on the breath (31). In severe DKA, there may be a decreased level of consciousness. DKA is a typical symptom of T1DM due to a complete lack of insulin production. T1DM patients become fully dependent on insulin therapy to survive. In the case of T2DM, these relative amounts of insulin are usually sufficient to suppress ketogenesis. If DKA occurs in patients with T2DM, their condition is called ketosis-prone type 2 DM (30).

T1DM is an autoimmune disorder associated with immune-mediated *β*-cell destruction. It is characterised by several immune markers, which are present in 85–90% of individuals with T1DM (32). These autoantibodies include islet cell autoantibodies (ICAs) to *β*-cell cytoplasmic proteins, insulin autoantibodies (IAAs), autoantibodies to islet-specific zinc transporter isoform 8 (ZnT8), glutamic acid decarboxylase autoantibodies (GADAs) such as glutamic acid decarboxylase 65-kilodalton isoform (GAD65) antibody, and autoantibodies to the tyrosine phosphatases, such as insulinoma-associated protein tyrosine phosphatase 2 (IA-2) (29, 32). These autoantibodies are gaining more clinical and diagnostic value in adults, with late onset of disease and slow progression of *β*-cell destruction, often being misdiagnosed as T2DM. In such cases, the presence of autoantibodies allows the correct diagnosis of the disorder as T1DM (29, 32).

Contrary to T1DM, type 2 diabetes is often associated with various lifestyle factors, such as age, family history of diabetes, poor diet, lack of exercise, and obesity (29). This form of DM commonly goes undiagnosed for many years as it progresses gradually and asymptomatically. In the early stages, the patient does not notice any classic symptoms of DM. Symptoms such as blurred vision, polyuria, or polydipsia are associated with advanced stages of the disease (32). Insulin deficiency and insulin resistance correlate with high levels of inflammatory cytokines and fatty acids in the plasma, leading to deficient glucose transport into target cells and increased hepatic glucose production. Overproduction of glucagon, along with insufficient insulin secretion to compensate for insulin resistance, causes high blood glucose values (25, 30). A large percentage of patients with T2DM are overweight or obese (9). Obesity influences the development of insulin resistance by releasing more free fatty acids to the liver, which increases hepatic gluconeogenesis (33). Under normal circumstances, when glucose levels rise, insulin signals adipose tissue to suppress the process of fat breakdown and use glucose sources in energy metabolism. Diabetic and obese patients exhibit overproduction of tumour necrosis factor *α* (TNF-α) and non-esterified fatty acids, which leads to reduced levels and dysregulation of insulin-signalling adapters, such as insulin receptor substrates (IRS) (33). The IRS are a family of proteins that are critical elements in insulin-signalling pathways (34). Disruption in IRS metabolism leads to insulin resistance (33, 35). Diabetics demonstrate an impaired suppression of adipose tissue lipolysis and an inability of insulin to inhibit hepatic glucose production, leading to chronic hyperglycaemia (33, 36). Patients with T2DM and accompanying obesity are at increased risk of developing macrovascular and microvascular complications (33, 37).

A critical factor in the pathogenesis and progression of DM and its associated complications is oxidative stress (38, 39). Cellular damage and dysfunction result from an imbalance between reactive oxygen species (ROS) production and the antioxidant defence mechanisms of the body (40). Increased ROS production due to chronic hyperglycaemia and mitochondrial dysfunction escalates oxidative stress after activation of metabolic pathways, including glucose autooxidation, with the enhanced formation of advanced glycation end products (AGEs), deactivation of the insulin signalling pathway, activation of the polyol pathway, hexosamine pathway, and protein kinase C (PKC) (38, 41, 42). This oxidative imbalance impairs *β*-cell function and suppresses insulin signalling pathways, driving insulin resistance (37). Prolonged oxidative stress causes dysregulation of glucose metabolism and contributes to increased inflammation (43, 44). The literature supports a strong correlation between hyperglycaemia and the formation and accumulation of AGEs (38, 39, 45). AGEs are heterogeneous particles derived from glycation, a non-enzymatic, random reaction between the carbonyl group of glucose and amino acids of proteins (45). The formation of AGEs is a complicated long-term molecular process known as the Maillard reaction (MR) (46). The first stage starts with the formation of non-stable Schiff’s bases, which subsequently rearrange into stable ketoamines known as Amadori products. Further chemical transformation of the Amadori products generates final molecules known as AGEs, which are responsible for alterations in cell signalling and functioning throughout the body (1, 45). This is due to specific receptors for advanced glycation end products (RAGE) found on many cell surfaces (46). In DM, AGEs’ interaction with RAGEs initiates various signalling pathways that contribute to the pathogenesis of many diabetic disorders, including vascular and skin complications (1, 45). AGE–RAGE interactions initiate inflammatory signalling pathways, such as nuclear factor kappa B (NF-κB), leading to the production of pro-inflammatory cytokines. Elevated levels of inflammatory mediators like TNF-*α*, IL-8, IL-6, IL-1β, and C-reactive protein (CRP) contribute to skin inflammation and immune-related skin disorders (47). Upon activation of RAGE, impaired wound healing and microbial infections are observed in DM skin (48). AGE accumulation in the epidermis results in the rearrangement of keratinocytes. The epidermis becomes thin, making the skin more susceptible to external damage (47). Additionally, the activation of the RAGE/NF-κB signalling pathway increases the release of matrix metalloproteinases (MMPs), especially MMP-1, MMP-2, and MMP-9, leading to collagen fibre deformation. AGEs form cross-links with collagen, altering the biomechanical properties of the fibres, making them stiff and less elastic (49). Some studies also reported macrophage dysfunction caused by the AGE–RAGE signalling axis (50). Macrophages are the main immune cells in the dermis involved in non-specific immune defence. High glucose levels promote the activation of macrophages, leading to an elevated synthesis of pro-inflammatory cytokines (51). Long-term exposure to hyperglycaemia, AGEs, and a chronic inflammatory state results in irreversible changes in cells (39, 52, 53).

## 3 Pathogenesis of diabetic neuropathy, retinopathy, and nephropathy

Chronic hyperglycaemia and the accompanying accumulation of AGEs, oxidative stress, and mitochondrial damage play a major role in the initiation of diabetic vascular complications, called vasculopathy (32). This general term refers to both microvascular and macrovascular complications. Diabetic microangiopathy is characterised by the proliferation of endothelial cells and the thickening of the basement membrane of arterioles, capillaries, and venules (6, 38). Neuropathy, retinopathy, and nephropathy are significant microvascular complications (5). Target tissues, such as nerves, the retina, and kidneys, exhibit heightened susceptibility to toxic glucose levels due to the distribution of glucose transporters (6).

Diabetic peripheral neuropathy (DPN) is a type of nerve damage connected with the progressive loss of nerve fibres (6). The clinical manifestation depends on the type of nerve damage. The most common forms of diabetic neuropathy include peripheral neuropathy, autonomic neuropathy, proximal neuropathy, and focal neuropathy (6). Sensory peripheral neuropathy predominantly affects the hands and lower limbs, especially the feet (54). Symptoms include tingling, numbness, burning sensations, weakness, and pain, resulting in loss of sensation throughout the body. In autonomic neuropathy, internal organs that control automatic functions of the body, such as digestion, blood pressure, and bladder function, are involved. Nerve damage in blood vessels results in altered blood flow regulation. Diminished sweating leads to dry skin, cracks, and fissures. Pain in the thighs, hips, or buttocks refers to proximal neuropathy, while weakness and sudden pain in the head or torso are connected with focal neuropathy (6, 54). The loss of nerve fibres begins distally in the lower extremities. Vascular alterations and the degeneration of distal nerve fibres result from poor repair processes and endothelial dysfunction (38). Hyperglycaemia contributes to the development of oxidative stress and overproduction of AGEs (38, 39, 45). This triggers chemokine and cytokine production, promoting inflammation and peripheral nerve fibre damage, which are responsible for conducting motor and sensory impulses (55). The progressively worsening condition of the lower motor neuron pathway is known as motor neuropathy. Motor neuropathy leads to significant disability, with loss of function in the feet, as well as reductions in muscular strength, mass, and flexibility (55, 56). In particular, damage has been demonstrated to the myelin sheath and Schwann cells, which play an important role in the development, maintenance, and regeneration of peripheral nerves. Impulse conduction and signalling disorders progress along the length, more often affecting the longest nerve fibres (56).

Diabetic retinopathy (DR) is an eye complication that causes damage to blood vessels in the retina and/or macula (6). Hyperglycaemia and the overproduction of AGEs play a key role in retinal capillary damage by initiating endothelial damage, capillary occlusion, aberrant blood vessel proliferation, retinal fluid leakage, and the appearance of microaneurysms (57–59). Chronic inflammation heightens vascular permeability and contributes to diabetic macular oedema, which is the most common cause of vision loss in patients with DR among diabetics (6). Persistent ischemia causes the release of proangiogenic factors like vascular endothelial growth factor (VEGF) (58). VEGF promotes the abnormal formation of new blood vessels and can generate serious complications such as vitreous haemorrhage or tractional retinal detachment (59, 60). Clinically, DR is divided into two stages: non-proliferative diabetic retinopathy (NPDR) and proliferative diabetic retinopathy (PDR). NPDR occurs first and refers to increased vascular permeability and capillary occlusion in the retinal vasculature (61). These pathologies, including microaneurysms, haemorrhages, and hard exudates, lead to the leakage of fluid and blood into the retinal tissue (62, 63). As DR progresses, symptoms like blurry vision, dark strings or spots in the field of vision, and gradual loss of vision occur. PDR is a more advanced stage of retinopathy and is characterised by the abnormal formation of new blood vessels in the retina. These vessels are weak and prone to breaking and bleeding into the vitreous, leading to vision impairment and blindness in an advanced state (6, 62).

Diabetic nephropathy (DN) or diabetic kidney disease (DKD) is a disorder characterised by persistent albuminuria (excretion of pathological quantities of urine albumin), diabetic glomerular lesions, a progressive decline in the glomerular filtration rate, and elevated arterial blood pressure (6). An injury to the highly specialised cells of the kidney glomerulus—podocytes—leads to albuminuria and chronic tubular injury (64). Symptoms include foamy urine, unexplained proteinuria, fatigue, foot oedema, and hypertension (6).

DPN, DR, and DN share common risk factors. Studies indicate that young age at onset of DM, followed by longer duration of diabetes, gender, elevated body mass index (BMI), dyslipidemia, or smoking correlate with an increased risk of many microvascular complications (6, 65–67). Moreover, the presence of neuropathy and nephropathy contributes to the development of retinopathy as a result of multiple vascular derangements in the body (8, 38). Therefore, effective management of these common risk factors is crucial to attenuate or delay the progression of microvascular disease in DM (6). Macroangiopathy in patients with DM proceeds in different phases, from endothelial dysfunction to low vessel wall elasticity and sclerosis (68). These changes result in cardiovascular system dysregulation with loss of elasticity of the vascular walls and peripheral circulatory failure (6, 7, 38). Recent studies indicate a correlation between diabetic macroangiopathy and diabetic polyneuropathy, emphasising the importance of metabolic changes and oxidative imbalance in the development of vascular dysfunction (8).

## 4 Epidermal, dermal, and adipose tissue abnormalities of the skin in DM

### 4.1 Skin structure and function

The skin is the largest human organ, consisting of the epidermis, dermis, and subcutaneous tissue. This multilayered construction is closely related to the functions of the skin. The skin serves as a constant interface between the external and internal environments. On the one hand, the skin protects the body from harmful external factors, but on the other hand, it ensures the reception of stimuli from the outside environment. Harmful agents that constantly interact with the skin include physical factors (heat, cold, or ultraviolet light (UV)), chemical factors (harmful acids and detergents), and biological factors (bacteria, viruses, and pathogenic fungi). The skin also regulates body temperature and prevents water loss (69, 70).

The most superficial layer of the skin is the epidermis. It consists of several distinct layers beginning with the innermost *stratum basale, stratum spinosum, stratum granulosum, stratum lucidum,* and *stratum corneum* (SC). The number of layers and overall thickness of the epidermis depends on the location in the body (69). Keratinocytes are the main cells of the epidermis. From the stratum basale, keratinocytes divide and differentiate to form new cells that move up to the skin surface to exfoliate. In healthy skin, the keratinocyte proliferation/differentiation balance ensures constant renewal of the epidermis, and it lasts 28–30 days (71). In SC, keratinocytes are terminally differentiated, anucleate, flattened, dead cells called corneocytes (72). Corneocytes, together with intercellular lipids (e.g., ceramide, cholesterol, and free fatty acids), form an effective outside-inside barrier, maintaining skin homeostasis and functions (73). Intercellular lipids in SC are end products delivered from the lamellar bodies of the epidermis—lipid granules in the granular layer. These structures are enriched in polar lipids, phospholipids, glycosphingolipids, free sterols, and catabolic enzymes, which are modified, rearranged, and hydrolysed to non-polar products that seal the junctions between keratinocytes (72). Glycosphingolipids are modified to ceramides while phospholipids are converted into free fatty acids (72, 74). In the meantime, keratohyalin granules—another structure in the stratum granulosum—begin to form keratins to fill the keratinocyte structure (74). During the terminal differentiation of epidermal cells, a highly phosphorylated protein in keratohyalin granules, called profilaggrin, is broken down into multiple filaggrin monomers (73). Further reactions lead to monomer degradation and the generation of a complex mixture of hygroscopic free amino acids, amino acid derivatives, and salts, which are components of the natural moisturising factor (NMF) (73). NMF constituents include serine, glycine, alanine, histidine, ornithine, citrulline, and arginine, as well as sodium pyrrolidone carboxylic acid (PCA), lactic acid, urea, and inorganic ions, such as potassium, sodium, magnesium, and calcium. These components are responsible for maintaining the water content of the SC by attracting and binding water molecules (73, 75). Released free amino acids into the cytoplasm initiate the aggregation of keratin filaments into tight bundles, stuck together by cross-linked molecules of other proteins, including loricrin and involucrin (73). The enzyme catalysing this process is transglutaminase 1, and the final products are corneal plates in the most superficial layer of the epidermis—flat, closely arranged corneocytes filled with keratin filaments (75). SC, intercellular lipids, and NMF constituents perform a physical and biochemical skin barrier.

The dermis is the second layer of the skin, connected to the epidermis by the basement membrane. The dermal-epidermal junction (DEJ) has a wavelike, undulating structure that is co-formed by epidermal protrusions down into the dermis and dermal elevations up into the epidermis (dermal papillae) (76, 77). That wavelike structure plays multiple roles in skin homeostasis and function, such as preventing delamination and ensuring the diffusion of nutrients from the dermis to the epidermis (76, 77). The dermis consists of fibroblasts (the main cells of the dermal connective tissue), collagen and elastin fibres, and ground substance, which is made of glycosaminoglycans (GAGs), with the most numerous being hyaluronic acid (76). Ground substances and fibre are components of the extracellular matrix (ECM) of the skin. The ECM maintains the correct hydration and structure of the connective tissue (77). The dermis is made up of two loose connective tissue layers: papillary and reticular. The papillary dermis is the upper portion beneath the epidermis, consisting of a small amount of collagen and a little fibre, but a large number of GAGs. The deeper reticular layer contains thick collagen and elastin fibres and creates an organised, compressed network, providing the proper strength and stiffness of the tissue. The dermis houses the hair, hair follicles, sweat glands, muscles, blood vessels, and sensory neurons (78).

The hypodermis, also known as subcutaneous tissue, is the innermost layer of the skin. It provides mechanical protection and thermal insulation, and it serves as the primary storage site for high-energy compounds. The hypodermis is composed of adipocytes—fat cells surrounded by connective tissue (77).

Sooner or later, patients with both types of DM present some cutaneous complications. As many as 70% of diabetes patients worldwide will develop cutaneous symptoms (79). A skin disease involves any medical condition that irritates or damages the human skin, hair, nails, and related glands and muscles. The dermatological manifestations of DM, attributed to hyperglycaemia, can have health consequences ranging from aesthetic concerns to life-threatening conditions (5, 11).

### 4.2 Epidermal barrier abnormalities in DM

Mechanisms underlying the altered epidermal permeability barrier function in DM are not clear and reveal conflicting findings. Some clinical studies demonstrate decreased SC hydration and transepidermal water loss (TEWL) in diabetic individuals, associated with a lack of glycaemic control and older patient age (79–81). These findings are confirmed by murine models, which indicate reduced levels of hyaluronic acid, decreased intercellular lipid synthesis, and lamellar body number as the main reasons for reduced skin hydration in association with increased blood AGEs (82). However, other clinical studies have not shown differences in SC hydration and TEWL in age- and gender-matched diabetic patients (79, 83). These contradictory results may stem from the presence of confounding factors, such as age and obesity, in the studied populations (79).

Several potential processes have been identified that contribute to the altered permeability barrier function in DM (72, 83, 84). These include reduced VEGF, antimicrobial peptides, and differentiation-related proteins, as well as increased skin surface pH and fatty acid content, with reduced overall epidermal lipid synthesis and psychological stress (84–86). Studies performed on keratinocyte cultures demonstrate that high glucose levels reduce the expression of VEGF and skin-derived antimicrobial peptides, such as *β*-defensin and cathelicidin (84, 87–89). These factors contribute to epidermal barrier homeostasis by regulating inflammatory responses, cytokine/chemokine secretion, cell migration, and proliferation. Disruption of these natural factors in DM leads to increased skin surface pH, reduced epidermal lipid production, as well as impaired keratinocyte differentiation and proliferation, ultimately resulting in delayed restoration of the permeability barrier (72, 84, 90). Recent studies show a significantly higher skin surface pH in mice and humans with T2DM, which may result from low sebum content in diabetic individuals (80, 84). Mouse models of T2DM have shown a reduction in overall epidermal lipid synthesis, with a concomitant increase in the content of short- and medium-chain fatty acids. Both conditions result in reduced permeability barrier function, and increased fatty acid content in the epidermis may further result in delayed restoration of the permeability barrier in diabetic patients (84, 91, 92). This may be due to reduced expression of loricrin and filaggrin in diabetic skin (73). Filaggrin is a granular and cornified layer protein, while loricrin is limited to the cornified layer of the epidermis (93). *In vitro* studies showed that high glucose levels inhibited the expression of loricrin and transglutaminase 1, which participates in the cross-linking and immobilisation of proteins in keratinocytes (84, 94). Therefore, reduced levels of differentiation-related proteins lead to delayed permeability barrier recovery (84, 95). Reduced expression of loricrin contributes to the overall fragility of the epidermis, increases the risk of infection, and delays wound healing. Transglutaminase alterations are also associated with wound healing disorders and inflammatory processes (84, 93). Finally, there is some evidence that psychological stress may contribute to decreased levels of antimicrobial peptide expression and epidermal lipid synthesis, which also adversely affect the epidermal barrier (84–86).

Several endogenous factors and different metabolic changes can contribute to reduced SC hydration levels in DM (83). First, in patients with DM compared to healthy controls, the content of skin surface lipids, which are supplied by sebum from sebaceous glands, is significantly lower (72, 83). Sebum is primarily composed of diglycerides, triglycerides, wax esters, squalene, cholesterol, and free fatty acids, and their reduced levels contribute to diminished skin hydration (96). Second, the content of SC intercellular lipids also decreases in diabetic individuals (83, 84). The level of ceramides, which are one of the major natural skin moisturisers, is reduced by over 60% in DM (84). Third, high concentrations of glucose inhibit keratinocyte proliferation and differentiation, as well as protein synthesis, which leads to disturbances in the production of cornified cells and NMF components (97). Finally, in the plasma of the diabetic murine model, hyaluronic acid levels are 25–70% lower compared to the control group. The possible reason is increased hyaluronidase activity in patients with DM, resulting in SC dehydration (84).

Epidermal barrier abnormalities in DM can provoke and exacerbate cutaneous inflammation (98). Reduced hydration of the SC in people with DM is a result of a disrupted skin barrier and leads to high levels of histamine and cytokines, as well as increased mast cell density, which are signs of skin inflammation (84). Patients with DM experience chronic itching, which may be exacerbated by high cytokine levels (99). Pruritus-caused scratching leads to further stratum corneum damage and disruption of the skin permeability barrier (83). In normal skin, the disrupted skin barrier is rapidly repaired, but in DM, recovery forces are delayed (79, 84).

### 4.3 Epidermal abnormalities in DM

In normal skin conditions, damage to the epidermal barrier causes the activation of keratinocytes and promotes the reepithelialisation process (100). During skin repair, keratinocytes undergo proliferation and migration, which is supported by reduced cell adhesion and proteolysis of ECM proteins by MMPs (79). Relative insulin deficiency in T2DM affects poor keratinocyte proliferation, differentiation and migration, resulting in impaired epidermal barrier function and contributing to the impairment of wound healing (100, 101). Excessive ROS in a high-glucose environment leads to increased activity of MMPs, especially matrix metalloproteinase-1 (MMP-1), matrix metalloproteinase-2 (MMP-2), and matrix metalloproteinase-9 (MMP-9) (102–104). MMPs play a critical role in suppressing keratinocyte migration, delaying wound healing (48). Under oxidative imbalance, inflammatory cells produce MMP-9, which selectively degrades the growth factors and other molecules that assist the healing process and modulate the expression of keratinocyte differentiation and migration (102–104). In keratinocytes, excess glucose levels escalate mitochondrial ROS overproduction, leading to mitochondrial oxidative damage (43, 105). Disturbance of mitochondrial membrane potential drives mtDNA fragmentation. Fragmented mtDNA alters signalling pathways, ultimately promoting an inflammatory response and keratinocyte apoptosis, which may delay diabetic wound healing (43, 106, 107). ROS such as nitric oxide have been found to have a strong regulatory effect on keratinocyte proliferation and differentiation (79, 100).

A recent study conducted on the skin of non-obese Sprague Dawley rats has provided insight into the multiscale characteristics of the skin of healthy and T2DM rats (108). Dwivedi et al. (108) report baseline data on the effects of T2DM on the physiological, structural, and mechanical properties of the skin. The physiologic stress–strain state (*in vivo* strain) was investigated, as well as the structural and mechanical response of the skin. Comparing healthy and diabetic animals, T2DM skin was found to be more susceptible to changes in mechanical response in terms of stiffness, transient stretch, anisotropy, and in vivo strain stress state (108). Mechanical anisotropy and in vivo strain were measured using a digital imaging correlation (DIC) technique and a DIC-coupled bulge experiment. Histology and fluorescence microscopy were used to evaluate the microstructure of collagen and elastin fibres, creating a constitutive model that considered the role of elastin fibres and the in-plane and out-of-plane distribution of collagen fibres. The obtained model was used to measure the state of *in vivo* stresses of healthy skin and skin with T2DM over the 360° planar directions (108). Morphological analysis at the epidermal layer level showed that, compared to healthy skin, epidermal thickness was significantly lower in T2DM skin. This makes the skin more susceptible to environmental aggression and trauma caused by mechanical stress. Furthermore, the wavy structure of the DEJ represented by dermal papillae almost disappeared in T2DM skin, leading to a reduction in DEJ length. The weakening of the attachment between the epidermis and dermis in T2DM leads to impaired skin sensation and nutrient delivery to the epidermis (108).

### 4.4 Dermal abnormalities in DM

Fibroblasts play a key role in the processes of ECM deposition and remodelling. On the one hand, they are synthetic cells that deposit a collagen-rich matrix, and on the other hand, fibroblasts are signalling cells that secrete growth factors to ensure cell–cell communication in the repair process (78). Fibroblasts incubated in a hyperglycaemic environment demonstrate a senescent phenotype and accelerated apoptosis (79). Any impairments in fibroblast function prevent normal ECM remodelling (79). Moreover, in DM, ECM proteins are subject to glycation-induced modification, resulting in the formation of AGEs (79, 109). Disturbed ECM remodelling is a typical symptom of wound healing failure and ulceration in patients with DM. During normal wound healing and ECM remodelling, damaged fibrils are degraded by ECM enzymes such as MMPs and replaced with newly synthesised and modified fibrils to regenerate the network (102, 109). The MMP family contains 23 members (110). ADAM and ADAMTS are two large metalloproteinase families involved in numerous cellular processes, including cell adhesion and migration, ectodomain shedding, and proteolysis. Collagen homeostasis is regulated by the MMPs and tissue inhibitors of metalloproteinases (TIMPs) (111). The balance between degradation and synthesis, which is maintained in the normal process, is disturbed in patients with DM (79). In defective wound healing, the production of more degraded, insoluble fibres predominates. It is accompanied by chronic inflammation and a highly proteolytic environment as a result of elevated levels of MMP-1, MMP-2, MMP-8, and MMP-9 (102, 112). TIMPs are natural regulators of the activity of MMPs (113). TIMPs selectively inhibit different MMPs as well as members of the disintegrin and metalloproteinase family with thrombospondin motifs (ADAMTS) (110, 114). The TIMP family consists of four members, from TIMP-1 to TIMP-4, each with subtly different protease inhibition profiles. TIMP-1, −2, and −4 are soluble inhibitors, while TIMP-3 is bound to ECM (110). The positioning of TIMP-3 in the matrix results from its interaction with sulfated proteoglycans of ECM, such as heparan sulfate (115). TIMP-1 strongly inhibits the activity of most MMPs; however, it is more limited in its inhibitory range than the other three TIMPs (110, 114). TIMP-1 binds particularly strongly to MMP-9 but has weak inhibitory properties against MMP-2, MMP-14, MMP-16, MMP-18, MMP-19, membrane type 1-matrix metalloproteinase (MT1-MMP), membrane type 2-matrix metalloproteinase (MT2-MMP), membrane type 3-matrix metalloproteinase (MT3-MMP), and membrane type 5-matrix metalloproteinase (MT5-MMP) (110, 116). TIMP-2 is the most abundant TIMP family member. TIMP-2 has been shown to interact with MMP-2 and MMP-14 (117). TIMP-3 has the widest inhibitory spectrum against MMPs, ADAM, and ADAMTS. TIMP-3 can suppress all MMPs, ADAMs (−10, −12, −17, −28, −33), and ADAMTS (−1, −2, −4, −5) (115). The MMP/TIMP imbalance in DM leads to ECM degradation and poor wound healing (102, 112). To confirm this assumption, a punch biopsy of wound tissue from chronic DM skin ulceration was performed. The results show increased expression of MMP-1, −2, −8, and −9, and decreased levels of TIMP-2 (111, 112). Pro-inflammatory cytokines, such as TNF-*α*, interleukin-1 (IL-1), and interleukin-6 (IL-6), may indirectly increase the production of MMPs (102). Continuous secretion of pro-inflammatory and fibrotic factors by tissues and cells under hyperglycemic conditions may be associated with the MMP/TIMP imbalance in diabetes (111). Increased levels of MMPs and accumulation of AGEs contribute to the degradation of collagen (109).

The MMP/TIMP balance in poor diabetic wound healing has been widely reported in the literature (111, 112). However, there are few reports on whether the MMP/TIMP ratio is imbalanced in early, intact diabetic skin, which could contribute to early intervention, clinical prevention, and treatment of skin lesions. Recent studies have provided knowledge that dermal collagen deposition disorders occur in diabetic non-injured skin (111). The skin of some DM patients before evident skin injury was stained with Masson’s trichrome. Results showed that dermal collagen was disordered and arranged in vague fascicles, and its density was variable. Collagen staining quantification and Western blot results show that the expression of collagen in DM skin was decreased. RNA sequencing performed on human dermal fibroblasts (HDF) under high glucose levels showed that the expression of *COL1A1* and *COL1A2* genes, which encode two alpha chains of type I collagen, was reduced (111). Additionally, the protein levels of collagen I in HDF cultures showed a decrease. This suggests that HDFs play an important role in collagen secretion in the skin of DM patients and that the collagen deposition disorder can be a result of decreased synthesis of new collagen or increased collagen breakdown. Moreover, an RNA-seq and qPCR analysis of the balance of MMP-2/TIMP-2 and MMP-9/TIMP-1, which can regulate collagen synthesis and decomposition, was disrupted in high glucose-treated HDFs, contributing to the skin collagen disorder in early, non-injured diabetic patients (111). The study also showed that after inhibition of MMP2 and MMP9 activity in mice with DM, the collagen deposition disorder was alleviated (111).

Dwivedi et al. (108) performed structural characterisation of the dermis in a non-obese T2DM rat model. The analysis shows a significant reduction in the areal density of collagen fibre in T2DM skin. In skin with T2DM, collagen fibres were fragmented and sparse; they also lost their arrangement and characteristics (d-periodicity), which results from a significant loss in relative protein content. In comparison, collagen fibrils in healthy skin are smooth, organised, and closely packed (118). An increase in average blood glucose levels affects the loss of collagen content due to an increase in MMP-1 and MMP-2 levels (108). The elevation of MMPs increases the breakdown and fragmentation of collagen, making the skin more prone to tears. In individuals with T2DM, collagen fibres in the skin lose their normal arrangement (e.g., dispersion and mean angle of orientation) and are aligned in only one direction, which can alter the orientation of the skin’s tension lines (44). This disruption contributes to impaired wound healing, making the skin susceptible to mechanically induced injuries, such as pressure ulcers. Elastin fibres in the T2DM skin model were also fragmented, which may impair the elasticity and regeneration of skin tissue (108).

### 4.5 Subcutaneous adipose tissue abnormalities in DM

**Figure 1:**
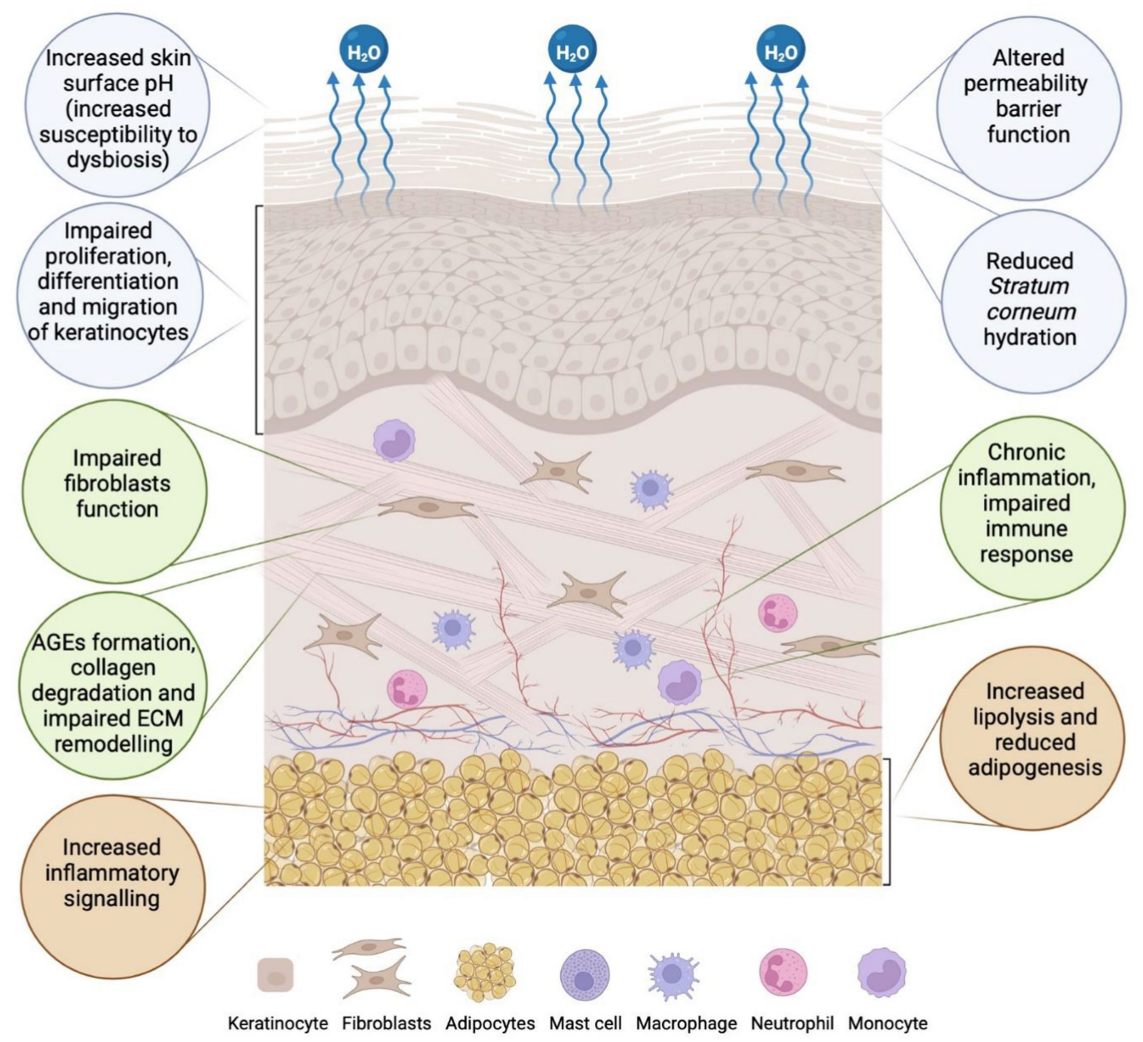
Epidermal, dermal, and adipose tissue abnormalities in the skin associated with diabetes mellitus (DM). In the epidermis (blue circles), factors that contribute to altered permeability barrier function include increased skin pH, reduced stratum corneum (SC) hydration due to water evaporation, and impaired proliferation, differentiation, and migration of keratinocytes. Changes in the dermis (green circles) result from impaired fibroblast function, the formation of advanced glycation end products (AGEs), collagen degradation, impaired extracellular matrix (ECM) remodelling, and chronic inflammation (43, 44, 107, 262). In subcutaneous adipose tissue (orange circles), changes such as increased inflammatory signalling, reduced adipogenesis, and increased lipolysis are observed (created in https://BioRender.com).

Patients with DM demonstrate signs of adipose tissue dysfunction within subcutaneous fat. These include enlarged adipocytes, increased inflammatory cytokines such as TNF-*α*, increased lipolysis, and reduced adipogenesis (79, 119, 120). There is evidence suggesting that adipose tissue dysfunction may precede the onset of DM, as shown in studies conducted on healthy individuals genetically predisposed to the disease (79, 119, 120). These studies demonstrated adipocyte atrophy and impaired differentiation, as well as increased inflammatory markers, such as IL1-*β*, IL-10, TNF-α, and early signs of adipose tissue remodelling and fibrosis (79, 121, 122). Adipose tissue is involved in cutaneous wound healing, which requires communication between adipocytes and macrophages—cells of the innate immune system. Adipocytes at the periphery of skin lesions promote the release of saturated and monounsaturated fatty acids to the wound surface. The presence of fatty acids ensures the activation of pro-inflammatory macrophages, accelerating vascular regeneration and skin wound healing processes (79, 123). It is also important to note that adipocyte-derived cells at the edge of the wound can differentiate into myofibroblasts (124). Myofibroblasts are primarily responsible for the production and maintenance of the ECM components during the proliferative phase of wound healing (79, 124, 125) (see Figure 1).

## 5 Diabetic angiopathy and neuropathy associated with DM

### 5.1 Diabetic foot ulcer (DFU)

DFU, including pressure ulcers and foot ulcers, are the most common complications in diabetic patients (11, 126–128). The WHO defined DFU as a set of symptoms that includes peripheral neuropathy, ischaemia from peripheral vascular disease, as well as infection of soft tissue and bone, manifesting as lower extremity ulceration and/or destruction of deep tissues (129).

Diabetic peripheral neuropathy, along with impairment of sensory, motor, and autonomic functions, makes the foot vulnerable to mechanical or thermal injury (8). With a reduced ability to feel pain, minor foot injuries may go undetected and develop into full-blown DFUs. Motor neuropathy disrupts the balance of biomechanical forces and foot anatomy, resulting in muscle atrophy and contractures (130). These pathogenetic events disturb walking motor skills, leading to poor balance and instability, as well as thickening of the skin in areas of chronic pressure, such as beneath the metatarsal heads (131). The horny epidermis presses on deeper tissues, facilitating ischemic necrosis and leading to the breakdown of skin and subcutaneous tissue integrity (132). In cases of decreased sweating, the skin on the lower limbs becomes dry and prone to cracks and fissures, with a predisposition to ulceration. Additionally, diabetic patients suffer from impaired wound healing, as hyperglycaemia reduces the effectiveness of healing mediators (102). Progressive autonomic neuropathy and atherosclerosis of the proximal arteries result in the formation of arteriovenous fistulas and foot ischemia, which impair the ability to heal properly (133, 134). Local osteomyelitis, dislocations, fractures, and significant disfigurement of the foot lead to Charcot foot arthropathy (128). Diabetic Charcot disease can affect one or more joints in the foot, leading to bone destruction and long-term deformities (128). Untreated DFUs are prone to secondary infection, which is accompanied by the presence of inflammatory and purulent lesions in or around the ulcer (130, 133). Data show that approximately 50% of ulcers become infected (130). Infection may spread to soft tissue, bones, and joints, leading to gangrene and lower limb amputations (52). It is very important to educate patients with DM about proper foot self-care and encourage them to wear adequately fitting and pressure-relieving footwear. As many as 42% of patients with healed DFUs will develop another ulcer within 1 year (130). To delay such a process, it is important to promote regular visits to a qualified specialist, called a podiatrist, to treat calluses and other forefoot symptoms (130).

Modern therapies for treating DFU are based on bioactive wound dressings. Among the wide range of ingredients, we can distinguish cellular and/or tissue-based products, placental dressings, 3D-bioprinted dressings, stem cell-based therapeutics, and acellular dermal substitutes (135). Bioactive dressings deliver various growth factors and maintain a moist wound environment (135). Equally standard are polymer-based wound dressings, which combine natural polymers (e.g., chitosan, cellulose) with synthetic polymers (e.g., polylactide, polyglycolic acid, polyurethanes). The properties of polymer dressings include swelling capacity, which provides a moist and warm environment to accelerate the wound healing process, excellent antibacterial and mechanical properties, and the ability to deliver bioactive substances (136).

**Figure 2:**
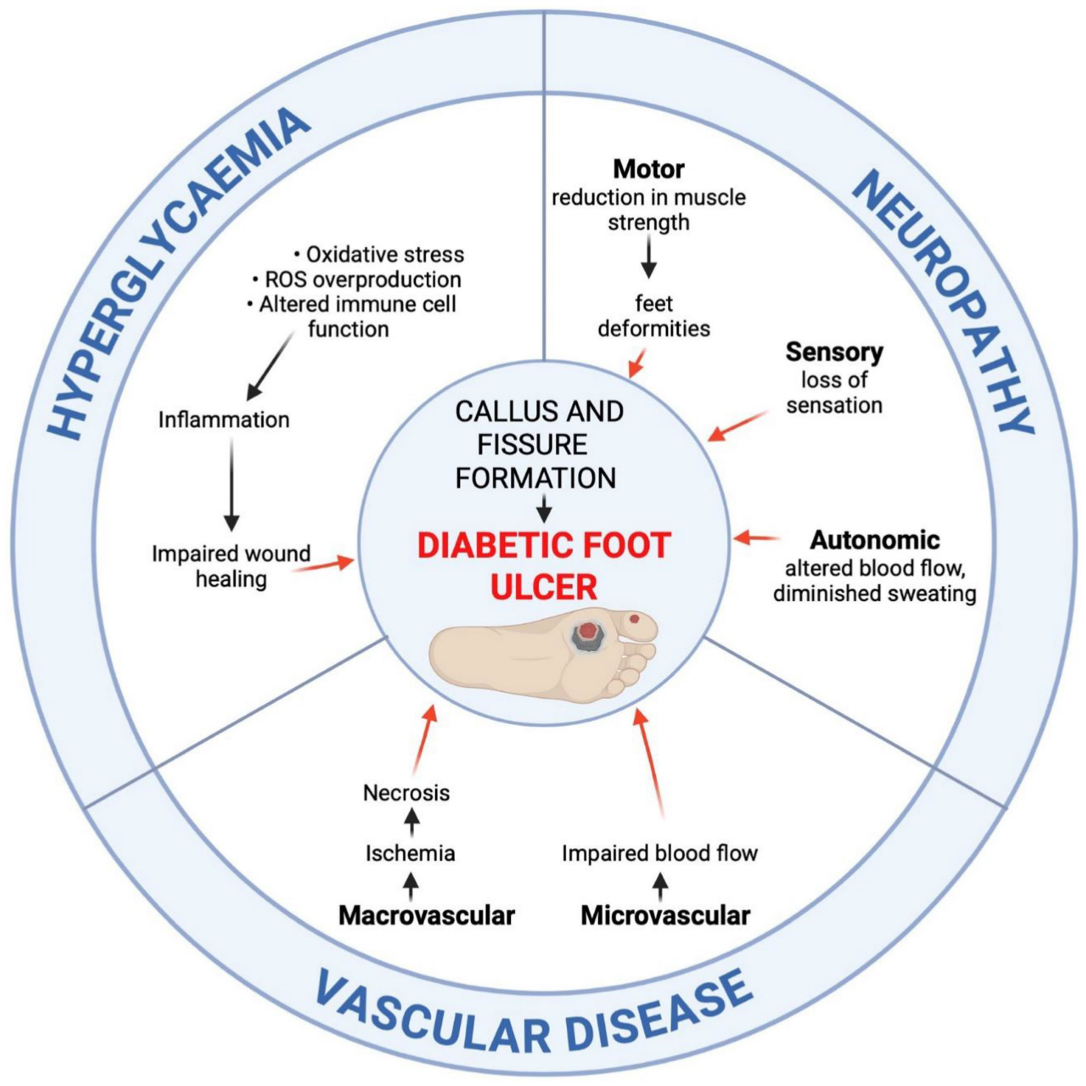
Developmental pathways of diabetic foot ulceration (DFU). The aetiology of DFU involves prolonged hyperglycaemia, peripheral neuropathy, and vascular disease. The prolonged hyperglycaemia impairs the wound healing process due to increased oxidative stress and reactive oxygen species (ROS) overproduction, altered immune cell function and inflammation, endothelial cell damage, impaired neovascularisation, as well as collagen cross-linking deformities. Peripheral motor, sensory, and autonomic neuropathy lead to foot deformities, decreased protective sensation, and skin dryness. Vascular disease accounts for the impaired blood flow, leading to ischemia and necrosis (created in https://BioRender.com).

DFU affects 15–25% of people with DM, with a higher incidence in patients with T2DM compared to T1DM (129). Apart from ulcers, other major diabetic foot complications include abscess, wet gangrene, dry gangrene, and necrotising fasciitis. As many as 75% of all cases of diabetic foot syndrome end in foot amputation (128, 137) (see Figure 2).

### 5.2 Diabetic gangrene

Reduced blood supply to the tissues of the foot, which leads to necrosis, is called gangrene. Gangrene is classified into dry, wet, and gas gangrene. Dry gangrene results from arterial occlusion, wet gangrene is more commonly associated with venous obstruction, while gas gangrene involves the production of gases by *Clostridium* bacteria (137).

In dry gangrene, dead tissue becomes numb, dry, dark, and shrunken. The ulceration is the starting point of necrosis, which spreads gradually, leading to surgical amputation or autoamputation (138). Spontaneous separation of unviable tissue from viable tissue is possible due to the occurrence of clear lines of demarcation (129, 139). Compared to surgical intervention, waiting for autoamputation may increase pain, induce secondary infection, and reduce the quality of life (128, 129). The pharmacologic approach for the treatment of dry gangrene involves the administration of antibiotics and painkillers, as well as circulatory management to improve blood circulation (137). Before any surgical decision is made, patients should first overcome peripheral artery disease. In medical management, the most promising therapy is antiplatelet therapy or platelet aggregation inhibitors (137). In wet gangrene, tissue is moist, swollen, soft, rotten, and dark. There is no clear-cut line of demarcation, and the putrefaction is notable due to the congestion of organs with blood. Wet gangrene results from obstruction or immobilisation of venous and/or arterial blood, leading to bacterial infection or sepsis. Wet gangrene spreads rapidly and can be fatal, so prompt surgical treatment is required (137). Gas gangrene is a life-threatening condition. In a hyperglycaemic environment, it spreads rapidly, with gas production at the infection site due to *Clostridium perfringens* bacterial infection (140). The presence of gas causes the tissue to turn pale, brown to purple-red with the development of multiple haemorrhagic blisters. Putrefaction is characterised by the infiltration of gases produced by bacteria in tissues, which spread rapidly to the surrounding areas. Radical amputation is the preferred treatment option (137).

### 5.3 Diabetic dermopathy

Diabetic dermopathy (DD) is a cutaneous manifestation of DM that often appears on the lower limbs, especially in the pretibial region over bony prominences (141). Some studies report that the prevalence of DD in the diabetic population exceeds 50%, especially in those with poorly controlled T2DM (142). Initially, DD is characterised by oval, dull, red papules that evolve over one to two weeks into atrophic, hyperpigmented patches and plaques with a fine scale (8, 141). It is believed that the pathogenesis of DD results from microangiopathic changes caused by hyperglycaemia, possibly in conjunction with mild trauma to affected areas, which leads to hemosiderin and melanin deposition in the skin (142). DD is a subtle, asymptomatic, and self-resolving clinical condition that does not require treatment (143). However, as a late complication of DM, DD reflects the progression of other diabetic microvascular complications, including retinopathy, nephropathy, and neuropathy (8, 144). An association with cardiovascular disease has also been reported (145). Therefore, the identification of DD is of significant importance to minimise the further progression of micro- and macrovascular complications (8, 142).

## 6 Skin manifestations strongly associated with DM

### 6.1 Yellow palms and soles

Patients with DM may experience a yellow discolouration of the palms and soles, known as carotenodermia (12, 146). Except for yellow pigmentation of the skin, this clinical condition is also associated with increased *β*-carotene levels in the blood. It has been reported that elevated serum carotene levels in diabetic patients are due to impaired conversion of pro-vitamin A carotenoids to vitamin A (147). Patients with hyperglycaemia consume a lot of vegetables and fruits with a high β-carotene content which can lead to hypercarotenaemia (147). However, yellowish discolouration of the palms and soles had developed only in 10% of cases (147). Unlike jaundice, carotenemia spares the sclera, which is useful in clinical differentiation (12).

#### 6.1.1 Acanthosis nigricans

Acanthosis nigricans (AN) is a highly prevalent dermatologic manifestation of DM and insulin resistance (11, 142). Clinically, AN is characterised by dark brown, velvety, lichenified plaques that are raised from the skin (148). It has a symmetrical distribution and is located in intertriginous areas such as the axilla, neck, and groin (144, 149). These lesions are usually asymptomatic, although itching may occasionally occur (8). The pathogenesis is thought to be due to persistently elevated blood glucose levels and the resulting state of hyperinsulinemia (11, 142). Insulin binding to insulin growth factor receptor 1 (IGF-1) on keratinocytes and fibroblasts induces cell proliferation, leading to the clinical manifestation of hyperkeratosis (9, 142). Changes in skin pigmentation are mainly due to the thickening of the SC of the epidermis and are less often due to changes in melanin production (9, 12). Apart from DM, AN is also associated with insulin resistance and obesity, and it can serve as a reliable cutaneous marker for these conditions (8, 9, 150). The most important therapy is the treatment of the underlying disease (142). The interventions for AN ultimately focus on reducing insulin resistance and improving glycaemic control through pharmacotherapy. Dietary modifications, increased physical activity, and weight reduction are promising lifestyle modifications that are helpful in overall therapy (150). Skin care procedures are based on keratolytic agents such as isotretinoin, salicylic acid, retinoids, or urea. Topical agents alleviate symptoms but do not eliminate the cause of the condition (142, 150).

### 6.2 Bullosis diabeticorum (BD)

Bullosis diabeticorum, or bullous disease (BD), is a rare skin manifestation affecting about 0.5% of diabetics (142, 144). Tense, non-inflammatory vesicles and bullae often occur on the hands and feet on an unchanged base. The diabetic bullae are large and painless, filled with clear fluid (143, 144). Blisters often appear rapidly and heal without scarring in 2 to 5 weeks (146). The fluid inside the blister is reabsorbed by the body, and the blisters dry up (143). Treatment for diabetic blisters is supportive and aimed at preventing secondary infection and chronic ulcers (11). To minimise the risk of infection, it is important not to puncture the blisters (143). The basis of therapy is the regulation of blood glucose levels (144). BD affects patients with long-duration DM or those who have diabetic microvascular complications (143). There is an incomplete understanding of the underlying pathogenesis of BD (12). It is assumed that the vascular complications of DM cause fragility of the skin, which promotes blistering. In addition, coexisting diabetic polyneuropathy may explain the foot involvement. There are also reports of BD appearing in individuals with prediabetes (8, 12, 142). Therefore, early detection of diabetic blisters may be an early marker of the disease (8).

### 6.3 Diabetic thick skin

Diabetic patients may have thickening and hardening of the skin on the dorsal aspect of the hand. The skin sclerosis on the extensor surface of the fingers, on the knuckles, or the periungual surface is known as Huntley’s papules (144). These are grouped, small, indurated papules, which may reduce joint flexibility (143, 146). Reduced joint mobility results in limited extension. Patients are unable to entirely close the gap between opposing fingers of closed hands (a “prayer sign”) (8). A scleroderma-like syndrome is common in T1DM and occurs in up to 50% of diabetic patients (146). The physiopathology of thick skin in DM is not completely understood. However, in a state of hyperglycaemia and hyperinsulinemia, collagen metabolism is disrupted (79). Increased collagen synthesis in fibroblasts and reduced degradation of collagen affect the thickening and hardening of the skin. There is no specific therapy for thick skin (12).

### 6.4 Scleredema diabeticorum

Another form of skin sclerosis associated with DM is the scleredema adultorum of Buschke (SAB). It is a rare connective tissue disease that affects mainly the face, trunk, neck, and upper limbs (146). It is characterised by painless, symmetrical, and diffuse thickening and hardening of the skin. Stiffness and impairment of mobility result from cutaneous deposition of collagen and mucopolysaccharides (12). Increased glucose levels stimulate collagen production from fibroblasts and reduce collagen degradation, affecting the thickening of the skin (8, 142). SAB is resistant to medical interventions. Therapies include glucocorticoids, pentoxifylline, prostaglandin E1, or methotrexate administration (146). However, to avoid the formation of new lesions, patients should monitor their blood glucose levels (12, 142, 146).

### 6.5 Necrobiosis lipoidica

Necrobiosis lipoidica (NL) is a chronic inflammatory granulomatous disease of the dermis. Initially, erythematous papules are present, which slowly evolve into a yellow-brown well-demarcated plaque with an atrophic centre (142). Lesions are typically present on the shins with no systemic symptoms (11). NL resolves spontaneously but frequently may develop secondary infection and ulceration (142). Treatment is challenging and typically involves topical therapy with corticosteroids and systemic immunosuppressants, such as cyclosporine and methotrexate (12). In recent years, cases have been reported of the successful use of ustekinumab and secukinumab, as well as Janus kinase inhibitors (JAKi) and the aryl hydrocarbon receptor agonist tapinarof (151, 152). Tacrolimus possesses anti-inflammatory and antifibrotic properties by inhibiting collagen synthesis (151). Although the aetiology of NL is considered unclear, histopathological examination indicates disorganisation and degeneration of collagen in the whole dermis and infiltration of inflammatory cells in the atrophic epidermis (146). Therefore, the use of tacrolimus can be a promising therapy (151). Autoimmune vasculitis appears to be a primary cause of collagen necrobiosis (146). In DM, prolonged hyperglycaemia causes microvascular ischemic changes affecting NL development (11, 146). There is a strong NL association with T1DM, with an incidence of 0.3 to 1.2% (8).

## 7 Non-specific symptoms associated with DM

### 7.1 Acrochordons (skin tags)

Acrochordons, known as skin tags or benign fibroids, are pedunculated, hyperpigmented, or skin-tone lumps that occur in diabetic patients (11). Approximately 23% of patients with DM have acrochordons (142). The neck, armpits, and periorbital area are most frequently involved (8). The pathogenesis of acrochordons includes a strong association with abnormal glucose metabolism and insulin resistance (8, 12). High insulin levels in response to hyperglycaemia stimulate keratinocyte proliferation and an increase in tissue and epidermal growth factors, resulting in the overgrowth of skin tags (9, 148). The changes are benign; therefore, they do not require removal for medical reasons. Aesthetic treatments include excision, electrotherapy, or cryotherapy (11). Interestingly, the quantity of acrochordons is positively correlated with blood glucose levels (11, 142). Studies indicate that the presence of 30 or more acrochordons in patients increases the risk of developing T2DM (142). Therefore, the presence and number of acrochordons may serve as a cutaneous marker for impaired carbohydrate metabolism (8, 9).

### 7.2 Rubeosis faciei diabeticorum

Rubeosis faciei is a chronic erythema of the face or neck of patients with DM (11). Telangiectasias, small dilated blood vessels near the skin surface, may also be seen. The redness of the skin is associated with diabetic microangiopathy and dilation of the superficial veins of the face (8). In addition, retinal vascular oedema contributes to the visual disturbances that often accompany patients with rubeosis faciei. This clinical manifestation occurs in up to 59% of hospitalised patients with DM (8). Since the underlying mechanism of rubeosis is microangiopathy, patients with DM should be carefully evaluated to exclude other concomitant microangiopathies, such as retinopathy or nephropathy (11, 153, 154). Treatment mainly involves glycaemic control (11).

### 7.3 Eruptive xanthomas

Eruptive xanthomas are another non-specific sign of DM, characterised by a sudden eruption of multiple reddish-yellow dome-shaped papules (146). They are located on the extensor surfaces of the extremities, buttock region, and hands (8). The pathogenesis involves a rapid formation of intracellular and dermal deposition of lipids as a result of hypertriglyceridemia (8). Uncontrolled DM is a common risk factor for triglyceride exacerbation (11, 146). Therapy for eruptive xanthomas consists of a proper diet or specific medication to control lipid metabolism (146). If medical therapy is ineffective, more invasive methods may provide improvement, such as laser therapy, cryosurgery, or surgical excision (11).

### 7.4 Acquired reactive perforating collagenosis

Acquired reactive perforating collagenosis (ARPC), or acquired perforating dermatoses (APD), is a rare skin manifestation of DM and chronic renal insufficiency (12). APD refers to a group of chronic skin disorders characterised by a loss of dermal connective tissue (12, 155). Histologically, perforating dermatoses result from an absence or degeneration of dermal connective tissue components, including collagen and elastic fibres (155). In diabetic patients, random glycation of skin proteins leads to disruption in collagen metabolism and hyperglycaemic complications in microvasculature (79). This may suggest the most likely pathogenesis of APD (79, 156). Clinically, patients present with erythematous papules or hyperkeratotic plaques with a centralised keratin plug on extensor surfaces of the arms and legs. The skin lesions are associated with pruritus (155). As a result of scratching and trauma to the epidermis, new APD lesions appear on areas of cutaneous injury, which is known as the Koebner phenomenon (12, 146). Treatment mainly consists of topical and oral retinoids or class II–III corticosteroids (amcinonide, desoximetasone, halcinonide, fluocinonide) (12, 157). In the last few years, allopurinol has also been reported as a good therapeutic option for ARPC (12).

### 7.5 Keratosis pilaris

Keratosis pilaris (KP) is a common benign condition of the skin’s hair follicles characterised by the appearance of pink-red monomorphic follicular papules (142). The characteristic lesions may appear on the outer sides of the upper arms, thighs, face, back, and buttocks (158). It is a common skin lesion in the general population, but the incidence and extent of lesions are greater in patients with T2DM (142, 158). In DM, hyperinsulinemia increases the level of circulating androgens, which drive hair follicle keratinocyte proliferation. This explains the association of hyperkeratosis in KP with DM (142). Keratosis pilaris can be treated with topical exfoliators, moisturisers, and emollients, but the most effective therapy is laser treatment (12, 158, 159).

### 7.6 Pruritus

Chronic pruritus is a common skin manifestation that occurs in diabetic patients, frequently caused by excessively dry skin (xerosis) (158). The dysfunction of sympathetic nerves, with impaired sweat function, is an important pathomechanism of skin dryness and hypohidrosis (diminished sweating) (12). In the case of diabetic polyneuropathy, sensory c-fibres are destroyed, which may also contribute to pruritus (158). The first step to enhance skin condition is the regular use of emollients and anti-pruritic substances, such as calamine (142). In more severe cases, it is necessary to use topical corticosteroids or even systemic antihistamines (11).

## 8 Other skin disorders associated with DM

### 8.1 Vitiligo

Vitiligo is an autoimmune pigmentary disorder is characterised by an absence or dysfunction of melanocytes (11, 158). It often affects the lower limbs, face, neck, and trunk (159). Vitiligo appears as scattered, well-demarcated areas of hypopigmented patches surrounded by healthy skin. It frequently occurs with other autoimmune disorders, including thyroid diseases and T1DM (146). Between 1 and 7% of T1DM patients manifest this skin alteration (146). In addition to autoimmune factors, it has been suggested that genetic and neurohormonal factors may also influence the development of vitiligo (12, 146). Damaged nerve cells release toxic substances that are harmful to melanocytes, leading to the destruction of these cells and a local lack of pigment. Infection or damage to the skin (Koebner phenomenon) may also contribute to the vitiligo (12, 159). Topical corticosteroids (betamethasone, fluticasone, hydrocortisone, clobetasol) are a satisfactory treatment for small and localised lesions, while treatment with ultraviolet B light is more effective for generalised vitiligo (146, 160).

### 8.2 Granuloma annulare

Granuloma annulare (GA) is a benign, non-infectious, and self-limited dermatitis. It is localised on the pretibial regions and extremities, particularly on the joints and dorsal hands and feet (12). GA is characterised by multiple pink-red papules of arciform and annular shape, with central, non-atrophic clearing (146). Initially, the lesions are small, firm, and skin-coloured, and they expand slowly in a centrifugal manner to form papules up to 5 cm in size (146). The lesions are usually asymptomatic and resolve spontaneously with central involution, resulting in hypo- or hyperpigmentation within 2 years (12, 146). The dermatological options include high-dose topical steroids, percutaneous injection of corticosteroids, PUVA therapy, or cryotherapy (12). Granuloma annulare can be localised or generalised, but the mechanism underlying the development of GA remains unclear (142, 146). Some studies indicate a correlation between generalised GA and T1DM, with a 10 to 15% prevalence in the diabetic population (142). It has also been reported that GA precedes the diagnosis of DM. Recurrent localised or generalised GA should prompt glucose testing to suspect DM (8, 142).

### 8.3 Lichen planus

Lichen planus is a mucocutaneous inflammatory condition affecting 25% of patients with DM (11, 144). Although the association is controversial, it has been reported that diabetic patients may also be at risk of developing oral lichen planus (11, 144). Clinically, it manifests as firm, erythematous, polygonal, pruritic papules with shiny, whitish streaks on the surface, called Wickham’s striae (11, 12). It usually affects the volar wrists and ankles, with possible involvement of the mucosa. New lichen planus lesions may be provoked mechanically (Koebner’s phenomenon) as a result of scratching the itchy areas (11, 12). There are several therapies for lichen planus. Topical or systemic corticosteroids, calcineurin inhibitors, phototherapy, or systemic retinoids (acitretin, etretinate) can be applied (12, 161, 162).

## 9 Cutaneous infections in diabetic patients

Patients with DM are more susceptible to developing skin and soft tissue infections (SSTI) due to several factors (8, 12, 52). Uncontrolled hyperglycaemia leads to metabolic and immunological alterations, making it harder to fight infection. As already mentioned, hyperglycaemia promotes oxidative stress in cells and the formation of ROS. It directly affects insulin signalling pathways and increases inflammation by activating pro-inflammatory cytokines (52). Diabetic neuropathy and angiopathy contribute to lower pain perception and unrecognised local mechanical trauma, leading to an increased risk of bacterial invasion (143). The skin pH in diabetic patients is higher, which provides a good environment for bacterial colonisation (52). An infectious episode will occur in more than 50% of patients with DM at some point during the disease (11). However, this risk of SSTI development seems to be higher in patients with worse DM control and higher glucose levels (8).

### 9.1 Bacterial infections

Disruption of the normal skin barrier in DM is an increased risk factor leading to bacterial invasion (163). In mild infections, the most frequently involved pathogens are Gram-positive cocci, including *Staphylococcus aureus* (8, 143). In deep tissue infections, Gram-negative organisms predominate, including *Pseudomonas aeruginosa* and *Enterobacteriaceae* (8, 52). Common bacterial skin infections in DM are folliculitis, abscesses, impetigo contagiosa, ecthyma, cellulitis, necrotising fasciitis, and erythrasma (11, 52, 143). While superficial infections are rather monomicrobial, in severe infections the aetiology is usually polymicrobial (8, 52). Overall, skin infections manifest with 2 or more clinical signs. Erythema, warmth, tenderness, pain, induration, purulent drainage, pustules, or boils are the most common lesions (11, 52). Recurrent bacterial skin infections should prompt examination for DM. Diabetic neuropathy and vascular complications, as well as altered immune function, are well-recognised risk factors in SSTI development (8, 12).

Folliculitis is an infection of one or more hair follicles. It is characterised by a tender, red spot, often with a surface purulent pustule (143). The condition may occur anywhere on hair-covered skin but is most common on the face, scalp, arms, and legs (12). People with DM may suffer from folliculitis due to a weakened immune system and poor circulation (12, 143). Treatment mainly involves topical antibiotic therapy (12).

Abscesses, including boils, are painful, red, swollen purulent bumps that can occur anywhere on the body. However, the most common localisation are the face, neck, armpits, buttocks, and thighs (164). Boils are a kind of deep skin infection caused by *Staphylococcus aureus* (143). Surgical treatment with pus and debris drainage and additional antibiotic therapy (clindamycin, trimethoprim-sulfamethoxazole) are the most satisfactory therapeutic procedures (12, 165).

Impetigo contagiosa is a superficial, highly contagious bacterial infection characterised by honey-coloured crusts and epidermal erosion (12). It affects the outermost layers of the epidermis and is typically caused by *Staphylococcus aureus* and *Streptococcus pyogenes* (12). Impetigo occurs individually or in clusters on the face or extremities. In the case of a single lesion, therapy with topical antibiotics is effective. Diffuse impetigo contagiosa should be treated with systemic penicillin (12).

Ecthyma is a skin infection caused by *β*-hemolytic group A streptococci and *Staphylococcus aureus* (12). Initially, skin lesions occur as macules with surrounding erythema but rapidly progress. They eventually take the form of small, brown-black, crusted sores, surrounded by erythematous and swollen demarcation (166). Ecthyma typically arises on the lower legs or feet. It is a deeper form of impetigo, as it causes erosions extending into the dermis (167). The crust that covers the ulcers in ecthyma is also thicker than the crust caused by impetigo. Effective therapy involves systemic antibiotics together with local antiseptics (12, 167).

Cellulitis is an extensive infection involving the dermis and subcutaneous tissue (143). β-hemolytic streptococci and methicillin-sensitive *Staphylococcus aureus* are the main causes of tissue infection (12, 168). Clinically, warm, brilliant erythema occurs with swelling, tenderness, and pain. Fever, impaired general condition, and leukocytosis may coexist (12). Appropriate targeted medication for this pathogen with systemic antibiotics (trimethoprim, sulfamethoxazole, clindamycin) is sufficient (12, 165, 168).

Necrotising fasciitis is a life-threatening streptococcal infection of the skin and the underlying tissue. Besides streptococci, it is triggered by *Staphylococcus aureus* and anaerobic bacteria (12). The clinical picture is dominated by early erythema, induration, and tenderness, which progresses to a severe painful haemorrhagic blister (169). Necrotising fasciitis is most commonly localised on the lower extremities (169). Urgent treatment includes extensive surgical debridement and systemic antibiotics. Life-threatening complications of necrotising fasciitis include thrombosis, sepsis, gangrenous necrosis, and organ failure (12, 169).

Erythrasma is a chronic superficial cutaneous disorder caused by a Gram-positive bacillus, *Corynebacterium minutissimum* (8). It is associated with the prediabetes stage when serum glucose levels have not yet reached a diagnostic value (170). Initially, erythrasma presents with non-pruritic, clearly demarcated, erythematous, and finely scaled patches that progress to brownish lesions with areas of central clearing and are slightly raised from the surrounding skin (171). These lesions are usually located in occluded groin folds, axillae, and gluteal cleft. The appearance and location of erythrasma can be easily mistaken for a fungal infection (8). The solution is Wood’s light and the differently coloured fluorescence phenomenon in each of these infections (8). The treatment of cutaneous erythrasma is based on oral, topical, and/or adjunctive therapies (171).

Diabetic Foot Infection (DFI) is a common and serious problem in diabetic people, which is often preceded by a DFU (52). Inflammatory symptoms include pus from a wound/ulcer, redness, swelling, pain, or warmth (52). However, due to peripheral neuropathy, signs of inflammation in patients with DM-related foot complications may be masked. DFI remains the most frequent DM-related complication and the most common cause of lower limb amputation (12). In a prospective study of diabetic patients suffering from a DFU, only 46% healed the ulcer (however, 10% had a recurrence), while 17% required lower limb amputation and 15% died (172). The selection of appropriate antibiotic therapy for the treatment of infected diabetic foot wounds requires taking into account the bacterial flora typical of this location, as most DFIs are polymicrobial (52). Diabetics are at higher risk of *Staphylococcus aureus*, including methicillin-resistant *Staphylococcus aureus* (MRSA), *Pseudomonas aeruginosa,* and MDR gram-negative bacilli (173).

### 9.2 Fungal infections

Candidiasis is a common fungal infection in diabetic patients (12). The most prevalent pathogen involved in cutaneous-mucosal candidiasis is *Candida albicans* (12, 174). Elevated glucose concentration and increased skin surface pH in the interdigital areas of diabetic patients promote an optimal environment for the development of Candida (143). In this case, the most frequent areas of candidiasis are interdigital areas (including erosion interdigital, balanitis), nails (including paronychia), and mucosa (including thrush and vulvovaginitis) (175, 176). Clinically, interdigital Candida infections manifest as a pruritic erythematous rash that progresses to vesicular-pustular lesions, and then to perforation and fissures (175–177). Nail candidiasis may present with periungual inflammation (paronychia) or subungual hyperkeratosis and onycholysis (8). Onychomycosis is a characteristic symptom in nearly one in two patients with T2DM and may be due to Candidal or dermatophyte infection (8, 143). Mucosal infection is characterised by the appearance of white papules and plaques, and erythematous erosions (8).

Infections caused by dermatophytes are also common in people with DM (8, 178). Skin dermatophytosis or onychomycosis is due to *Trichophyton rubrum* and *Trichophyton interdigitale*, which are the most prevalent dermatophytes in this condition (12, 179, 180). Mycosis can affect various areas of the body, but tinea pedis (foot) is the most common dermatophyte infection in diabetic patients (8, 179). Clinically, it is manifested by erythematous, horny, or bullous lesions with itching or pain (180). If not treated hastily, relatively benign dermatophyte infections can lead to serious consequences, such as secondary bacterial infection (8). The treatment consists of topical or systemic antifungal medications (181).

**Figure 3:**
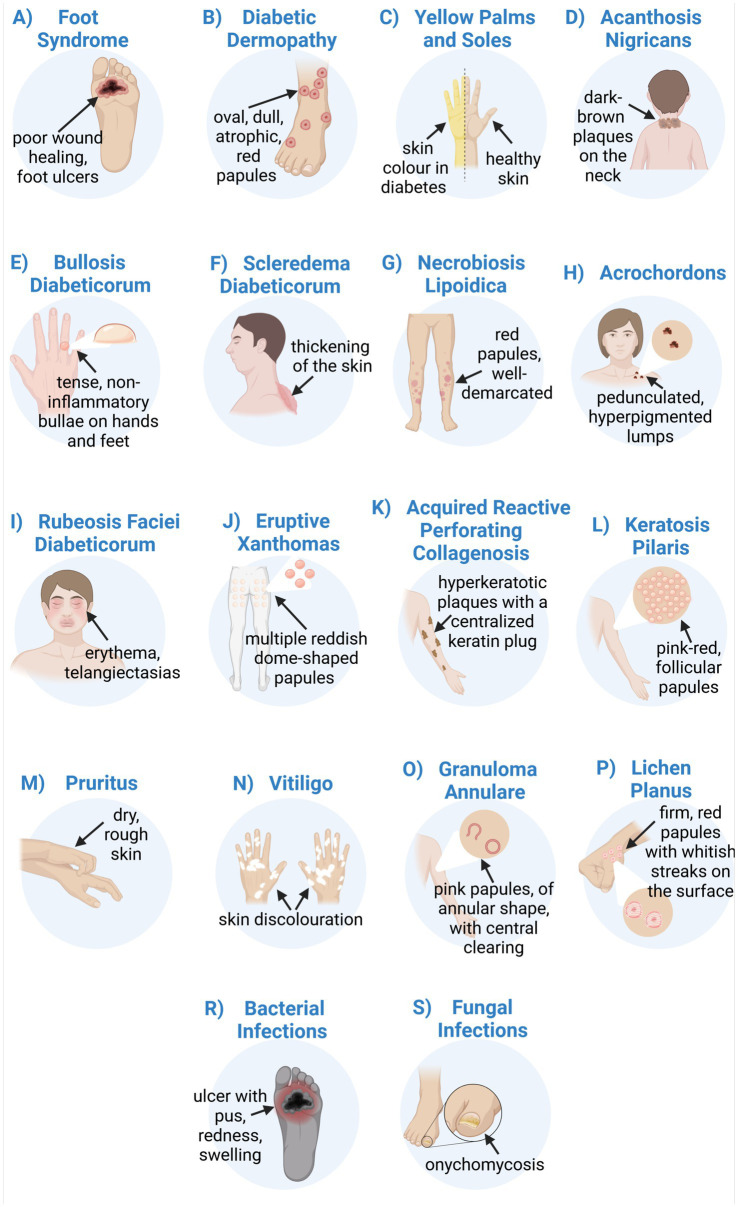
Skin manifestations as a cutaneous marker of diabetes mellitus (DM). **(A)** Foot syndrome is connected with impaired wound healing, diabetic foot ulcer (DFU), and dry or wet gangrene. **(B)** Diabetic dermopathy manifests as oval, dull, red papules, atrophic, hyperpigmented patches, and plaques with a fine scale. **(C)** Yellow palms and soles. **(D)** Acanthosis nigricans, dark-brown, velvety lichenified plaques that are raised from the skin. **(E)** Bullosis diabeticorum, tense, non-inflammatory vesicles, and bullae. **(F)** Scleredema diabeticorum and thick skin refer to painless symmetrical and diffuse thickening of the skin with reduced joint mobility. **(G)** Necrobiosis lipoidica manifests as erythematous papules and a well-demarcated plaque with an atrophic centre. **(H)** Acrochordons, or skin tags, are pedunculated hyperpigmented lumps. **(I)** Rubeosis faciei diabeticorum—erythema, vascular oedema, and telangiectasias. **(J)** Eruptive xanthomas are connected with multiple reddish-yellow dome-shaped papules with a tendency to sudden eruption. **(K)** Acquired reactive perforating collagenosis is accompanied by pruritus, erythematous papules, and hyperkeratotic plaques with a central keratin plug. **(L)** Keratosis pilaris—a pink-red monomorphic, follicular papules. **(M)** Pruritus/dry skin/xerosis. **(N)** Vitiligo, known as skin discolouration. **(O)** Granuloma annulare manifests as multiple, pink-red papules up to 5 cm in size, of arciform and annular shape, with central non-atrophic clearing. **(P)** Lichen planus, a firm erythematous polygonal pruritic papule with shiny whitish streaks on the surface. **(R)** Bacterial infections include folliculitis (pustule in hair follicles), abscesses (painful red swollen purulent bumps—boils), impetigo contagiosa (honey-coloured crusts and epidermal erosion), ecthyma (small brown-black crusted sores with surrounding erythema), cellulitis (warm tenderness, brilliant erythema, fever of skin tissue), necrotising fasciitis (early erythema progresses to a severe painful haemorrhagic blister), erythrasma (clearly demarcated erythematous lesions turn into brownish with central clearing raised from the skin), diabetic foot Infection (wound/ulcer with pus, redness, swelling, pain, or warmth). **(S)** Fungal infections include candidiasis (pruritic erythematous rash, vesicular-pustular lesions, perforation, and fissures), dermatophytosis (erythematous, horny, or bullous lesions with itching or pain), onychomycosis (white papules and plaques, and erythematous erosions), mucormycosis (sinusitis with purulent nasal discharge, rash, facial erythema, oedema, and cellulitis with systemic fever) (created in https://BioRender.com).

A rare and lethal disease called rhinocerebral mucormycosis can occur due to a fungal infection of the otorhinolaryngological tract (12). *Rhizopus oryzae* is a common pathogen responsible for mucormycosis (182, 183). The infection starts from sinusitis with purulent nasal discharge, which progresses to a rash, facial erythema, and oedema, and then to cellulitis with systemic fever (12, 144). Rhizopus affects nerves and vessels, causing numbness and vascular necrosis, which manifests in the nasal or palate mucosa (182). The infection may evolve, leading to extensive necrosis and thrombosis (183, 184). Mucormycosis requires urgent treatment with surgical necrotic debridement and intravenous administration of amphotericin B (12). A total of 31% of cutaneous infections and 62% of rhinocerebral infections result in the patient’s death (182). Interestingly, rhino-orbito-cerebral mucormycosis and pulmonary mucormycosis are common forms of COVID-19-associated mucormycosis (185). Poorly controlled blood sugar levels and immune dysregulation increase the risk of mucormycosis among COVID-19 patients. Indeed, COVID-19 predisposes to opportunistic fungal infections by reducing the number of T lymphocytes, CD8 + T cells, and CD4 + T cells. The use of steroids in COVID-19 therapy may therefore exacerbate carbohydrate and immune disorders (185, 186) (see Figure 3).

## 10 Skin complications due to therapy of DM

### 10.1 Cutaneous reactions to insulin

The classical continuous adverse effects of insulin application include lipoatrophy, lipohypertrophy, as well as subcutaneous nodules, local infections, and insulin allergy (11, 144, 187).

Lipoatrophy at the site of insulin injection is characterised by a loss of local subcutaneous fat and occurs as a small dent at the injection site (188). The pathological mechanism involves activation of an inflammatory cascade in the adipocytes, which is a response to vascular deposits of immunoglobulins (12, 189). It was found that lipoatrophy is associated with the method of insulin administration and the type of insulin. The introduction of purified insulin led to a reduced incidence of lipoatrophy (12, 188). Lipoatrophy could be treated with oral corticosteroids, as this can induce the differentiation of adipocytes. Promising evidence in the therapy of lipoatrophy showed significant improvement with betamethasone injection (188).

In contrast, approximately 27% of people with DM may develop lipohypertrophy (12). It is defined as adipocyte hypertrophy and an increase in local subcutaneous fat. Clinically, it manifests as soft cutaneous nodules resembling lipomas of variable size (12, 190, 191). The physiopathology is probably associated with insulin-dependent activation of adipocytes (12, 190). Another common cutaneous symptom of insulin application is a bacterial infection (12, 192). The number of daily insulin injections is positively correlated with the risk of local bacterial infections (193). Lipohypertrophy normally improves over a few months after discontinuing injections at that site (12).

Insulin allergy is rare but challenging for diabetic patients. Insulin hypersensitivity occurs in 0.1–3% of people with DM, and clinical symptoms depend on the immune mechanism involved (194). Allergy may range from cutaneous reactions, which are either immediate (type I, IgE-mediated) or delayed (type IV, T-cell-mediated), to less frequent generalised reactions (194, 195). Immediate skin manifestations at the injection site include pruritic urticarial papules, while delayed reactions are described as subcutaneous inflammatory nodules, with temporary itching or pain (193, 196). Systemic manifestations refer to life-threatening anaphylaxis and angioedema (197). Suspicion of an allergy to insulin injections requires the detection of specific IgE antibodies to insulin in the patient’s serum or plasma, as well as skin tests (skin prick tests or intradermal tests) (12, 198). Treatment of cutaneous allergies to insulin involves using medicines such as antihistamines (cetirizine, desloratadine), leukotriene inhibitors (montelucast), and topical steroids (methylprednisolone, hydrocortisone) (194, 197).

### 10.2 Cutaneous reactions to oral antidiabetic agents

Reactions to oral antidiabetic drugs are rare but can induce cutaneous adverse reactions (CADRs), which most commonly manifest as phototoxic or photoallergic drug eruptions, erythema multiforme, leukocytoclastic vasculitis, psoriasiform eruptions, lichenoid drug eruptions, or pemphigus vulgaris (11, 187, 199).

Metformin is a first-line oral agent for the treatment of T2DM, which is a suppressor of hepatic gluconeogenesis (25). The most commonly reported CADRs after metformin therapy are leukocytoclastic vasculitis (LCV) and psoriatic drug eruptions (11, 187, 200). LCV consists of haemorrhagic lesions, both papules and bullae, caused by capillaries and venules, while psoriatic eruptions present as scaly 3 plaques, sharply demarcated, found on the extensor surfaces (187). However, metformin has a generally good safety profile, and CADR incidents are very rare (15).

The drugs belonging to the sulphonylurea class, such as glibenclamide, tolbutamide, and chlorpropamide, are another group of oral antidiabetic drugs recommended when metformin is intolerant or ineffective (199). CADRs occur in about 1% of diabetics using sulfonylureas and include non-specific reactions of photosensitivity –phototoxic or photoallergic drug eruptions (199, 201). Photosensitivity reactions occur after exposure to a photosensitising drug and either ultraviolet (UV) or visible radiation. Photoallergic drug eruptions result from an immune-mediated mechanism of action and clinically manifest as slightly itchy erythema and eczema eruption (202). However, phototoxic drug eruptions, which are not immune-mediated, are much more frequent (202). They result from direct cellular damage when sufficient doses of the drug and radiation are present. Phototoxic drug eruptions manifest as excessive sunburn reactions with erythema, itching, and a burning sensation (11, 202). Some studies suggest that sulphonylurea drugs are involved in psoriatic lesions, lichenoid eruptions (symmetric, erythematous, violaceous papules similar to lichen planus), or pemphigus vulgaris (blisters on cutaneous and mucosal surfaces) (203, 204). Serious life-threatening mucocutaneous reactions, such as Stevens-Johnson syndrome and more severe toxic epidermal necrolysis, have also been documented, characterised by blisters and skin detachment (187).

Acarbose is an alpha-glucosidase inhibitor and a useful antidiabetic drug for patients at high risk of hypoglycaemia after sulfonylurea derivatives and metformin. Among CADRs after acarbose intake, a case of erythema multiforme was reported. These are erythematous plaques with vesicles all over the body (199, 205).

## 11 Therapeutic management of DM

Metabolic control in DM is a critical component of DM care. Proper management and control of glycosylated haemoglobin (HbA1c), LDL cholesterol, and blood pressure are key to preventing complications and reducing the risk of mortality (206, 207). A long-term study on patients with T1DM showed comparable mortality outcomes in the intensive treatment group (mean HbA1c 7% [53 mmol/mol]) with the general American population (mean HbA1c 6.5% [48 mmol/mol]), demonstrating the importance of metabolic control in DM (208). Another study showed that a difference of approximately 0.9% in HbA1c values translated into a 13% lower risk of death in patients with T2DM (208). Patients with T2DM often exhibit a disturbed lipid profile (209). Lipid abnormalities, often termed diabetic dyslipidemia, include high plasma levels of total cholesterol (TC), triglycerides (TG), low-density lipoprotein cholesterol (LDL-C), and free fatty acids, with decreased levels of high-density lipoprotein cholesterol (HDL-C) (209). Controlling LDL-C with a statin provides a mortality benefit. Data suggest that lowering LDL-C levels by 38.6 mg/dL (1 mmoL/L) each reduces mortality by approximately 9% in DM cases (208). Blood pressure control is another major determinant of mortality in DM. As data suggest, each 10 mmHg lower systolic blood pressure translates into a 13% lower risk of death in patients with T2DM (208). Achieving normoglycaemia and maintaining correct parameters of disease compensation after medical treatment may improve the overall quality of life and reduce the risk of future complications (206). A growing body of literature highlights metabolic imbalance as a key driver of skin symptoms in DM (210–212). A recent study found a strong positive association between inadequate HbA1c levels and elevated inflammatory markers—C-reactive protein (CRP), IL-6, and TNF-*α*, and skin disease severity in patients with DM (212). This multivariate analysis showed that patients with the highest HbA1c levels and long duration of diabetes showed the most severe dermatological symptoms. The study also indicates the effect of improved metabolic control on skin symptom severity over 24 months. With a decrease in HbA1c levels from 9.2% at the beginning of the study to 6.5% after 24 months, skin severity values fell from 8.5 to 3.2 over the same period (212). Substantial improvement was marked especially in diabetic dermopathy, necrobiosis lipoidica, and acanthosis nigricans. Tight glucose control and early intervention on signs of inflammation may improve dermatological outcomes in DM patients (212).

Achieving better glycaemic control in patients with diabetes requires the implementation of antihyperglycaemic drugs. The number of antihyperglycaemic drugs available is constantly increasing, but the most effective pharmacological agents are metformin and insulin. Metformin is an oral first-line drug for the treatment of T2DM, while insulin can be used successfully for both T1DM and T2DM (25, 213). Treatment regimens and therapeutic goals should be individualised, aiming to improve hyperglycaemia and reduce the risk of micro- and macrovascular complications, taking into account comorbidities, body weight, and the potential impact of drugs on the development of hypoglycaemia. Metformin should be especially considered for DM prevention in adults with a BMI ≥ 35 kg/m2, age ≥ 60 years, and elevated fasting plasma glucose (≥ 110 mg/dL) (15). Recent studies showed promising effects of metformin in combination with dipeptidyl peptidase 4 (DPP-4) inhibitors and glucagon-like peptide-1 (GLP-1) receptor agonists (4, 214–216). GLP-1 can increase insulin production in the pancreas and suppress appetite, resulting in improved tissue insulin sensitivity and weight loss, which is helpful in the treatment of obese patients with T2DM (15). T1DM patients require lifelong insulin therapy. Only 20–30% of patients with T2DM with progressive pancreatic *β*-cell dysfunction require insulin therapy (213). Insulin treatment includes long-acting or intermediate-acting insulin analogue injections for prandial glycaemic control. Therapeutic insulins have been classified by generation to highlight the clinically relevant characteristics of various insulin preparations, based on concentration, glycaemic management, and approximate time–action profile (213). However, the medical treatment of DM causes several systemic changes, including skin alterations. As already mentioned, lipoatrophy, lipohypertrophy, subcutaneous nodules, local infections, or insulin allergy are the most common skin symptoms of insulin therapy (11, 12, 188). On the other hand, oral metformin contributes to the progression of phototoxic or photoallergic drug eruptions, erythema multiforme, leukocytoclastic vasculitis, psoriasiform and lichenoid drug eruptions, or pemphigus vulgaris (11).

It is well known that lifestyle interventions, such as a healthy diet and regular physical activity, can positively influence the course of the disease (206). According to the latest knowledge, an intensive lifestyle intervention targeting weight loss can reduce the incidence of T2DM in overweight/obese patients with impaired glucose tolerance by 58% over 3 years (217). Proper nutrition in DM ensures control of glycaemia, body weight, and improvement of cardiovascular risk factors such as blood pressure and lipid profile. The dietary modification consists of a reduced-calorie meal plan. A daily energy deficit of 500 kilocalories is essential for effective weight loss (217). In patients with DM, Mediterranean diets, low-fat diets, low-carbohydrate diets, vegetarian diets, and vegan diets have been shown to be effective for weight loss and maintaining stable glycaemia (217). The gold standards for nutrition in DM are consistency in daily carbohydrate intake, limiting the intake of high glycaemic index or sucrose-containing foods, avoiding foods with added sugars, fats, and sodium, and ensuring adequate protein intake and timing of meals (217–219). To improve overall health, daily nutrition should emphasise a variety of nutrient-rich foods in adequate portions, rich in dietary fibre, vitamins, and minerals (218). A healthful eating plan provides sufficient micronutrients, and routine supplementation of vitamins and minerals is not necessary. There is no evidence that the intake of magnesium, vitamins A, C, and E, cinnamon, curcumin, or *aloe vera* supplements improves glycaemia (217, 218, 220). Only vitamin B12 deficiency in patients treated with metformin is supported by evidence (217, 218, 220). High-dose oral B12 supplementation may be effective in restoring normal glucose levels. In addition, it has been shown that supplemental chromium can decrease fasting glucose levels and improve glucose tolerance (218). The dietary conditions mentioned above have a positive impact on the overall health of patients with DM. However, no rigorous studies confirm the positive effects of a healthy diet on skin conditions in patients with DM. To support the impact of dietary habits on improving skin conditions, another inflammatory skin disease—psoriasis—will be mentioned. Psoriasis is often comorbid with metabolic syndrome, which refers to the co-occurrence of several cardiovascular risk factors, including T2DM or insulin resistance (221, 222). There is a cross-sectional study that relates healthy dietary interventions with skin improvement among patients with psoriasis (221). As it turns out, a diet low in alcohol, gluten, and nightshade vegetables but rich in fish oil/omega-3, vegetables, and vitamin D contributes to relieving psoriasis symptoms and reducing skin inflammation (221, 223). Recent studies also point to the antiglycoxidant properties of vitamin D (224). Moreover, studies have shown a favourable skin response following the Pagano diet (which involves decreased intake of nightshades and processed foods for the benefit of an increased intake of fruits and vegetables), vegan diet (based on plants), and Paleolithic diets (with fresh vegetables and fruits, lean meat, nuts, and olive oil) (221, 223, 225). A diet rich in animal products increases the intake of saturated fatty acids and trans fatty acids. Eliminating meat from the daily diet and increasing consumption of vegetables, fruits, legumes, and nuts will provide anti-inflammatory components such as antioxidants and omega-3 fatty acids, which will benefit psoriasis skin lesions (221). Antioxidant components in dietary foods such as tea, coffee, wine, herbs (including thyme, rosemary, mint, parsley, basil, and oregano), oils (such as olive oil and avocado oil), and honey may help alleviate T2DM. Their antidiabetic action is based on maintaining glucose homeostasis, regulating insulin secretion, and increasing tissue sensitivity to insulin (226, 227). A comprehensive awareness of the therapeutic potential of antioxidant food components can lead to better management of chronic diseases, including diabetes. Dietary antioxidants can be a component of alternative treatment or in combination with drug therapy (228, 229). A cutting-edge strategy of skincare is based on integrating dietary solutions and topical components. The association of active ingredients from dietary sources, such as antioxidants and vitamins, with topical peptides or antioxidants is a holistic method to improve skin health and rejuvenation in the most common dermatitis (230–232).

Regular physical activity can prevent T2DM progression (218). Increasing moderate-intensity physical activity and aerobic exercise to at least 150 min per week is required to achieve and maintain 7–10% of initial weight loss in obese patients (217, 218). The benefits of intensive lifestyle interventions and weight-loss procedures in people with T2DM have been thoroughly investigated in a randomised trial of 5,145 people (217). The subjects followed a low-fat and low-calorie diet ranging from 1,200 to 1800 kilocalories per day, depending on their initial body weight. Moderate-intensity physical activity, similar to brisk walking, of at least 175 min per week was introduced. Such an intensive lifestyle intervention resulted in a weight loss of approximately 8.6% after 1 year and 4.7% after 4 years. This was accompanied by lower blood sugar, less need for DM medication, an improved lipid profile (higher HDL-C levels, lower triglycerides), lower diastolic and systolic blood pressure, and remission of DM in around 10% of people (217).

### 11.1 Skin care in DM

Appropriate care for skin with diabetic complications includes preventing, detecting, and managing skin lesions. Unfortunately, skin disorders constitute a serious aesthetic problem, often impossible to hide. Thus, to improve skin health and function, people with DM must have adequate knowledge, self-efficacy, and proper skin care habits (15, 143). Diabetic patients demonstrate epidermal dysfunction, including disruption of the permeability barrier, reduced stratum corneum hydration, and increased skin pH (72, 84, 85, 90). All these conditions promote cutaneous inflammation and reduce comfort and quality of life (15, 233, 234). Maintaining skin integrity and preventing skin complications are the gold standard in diabetic skin therapy. The International Diabetes Federation, in agreement with the American Diabetes Association, has established guidelines on the prevention and management of diabetes-related skin complications (143). These protocols provide an optimal knowledge resource for healthcare providers and diabetic individuals. It also emphasises the importance of regular skin assessments during routine DM care. DM skin symptoms are often the first sign of uncontrolled blood glucose levels. Early detection can prevent systemic disease progression and allow timely intervention (143). Adherence to treatment recommendations is a major challenge for people with DM (158). Education about proper skin care practices includes the importance of daily cleansing, moisturising, skin inspection, maintaining good hygiene, and drying areas prone to excessive moisture, such as the feet (143, 158).

Recent studies have shown an improvement in inflammatory skin conditions following a combination of plant extracts, peptides, and antioxidants (230, 235–237). Peptides like palmitoyl tetrapeptide-7 and palmitoyl tripeptide-1 provide proper hydration, stimulate collagen and elastin production, reduce inflammation, and promote skin repair, which could be successfully used in DM skin (230, 238). Moreover, copper peptides in topical applications promote wound healing (230). Several studies have proven the effectiveness of natural extracts against chronic wounds and skin infections (239). In a holistic approach, a combination of different mechanisms of action of substances is required to achieve an overall improvement in skin condition (230, 240). Therefore, a combination of marine collagen peptides and plant-derived antioxidants (coenzyme Q10, grape skin extract, luteolin, selenium) provides better results in skin properties (241). Antioxidants in skin care formulations, including vitamins C and E, carotenoids, and resveratrol, can be successfully combined with dietary antioxidants or probiotics for better protection against inflammatory-induced damage (239). Natural extracts, for example, green tea extract, combined with dietary and topical probiotics, inhibit lipid peroxidation and MMP activity and provide enhanced antioxidant defence mechanisms (230, 239).

To maintain the integrity of the skin barrier and prevent dry, itchy, or scaly skin, patients with DM need to use moisturisers, called emollients, in their daily care (158, 242). Emollients are classified into: (1) superficial moisturisers, such as collagen, hyaluronic acid, and chitosan, (2) humectants, binding water in the SC, for example, glycerol, glycols, panthenol, sorbitol, mannitol, urea, (3) occlusive ingredients which form a barrier on top of the skin and prevent water loss, such as lanolin, eucerin, phospholipids, paraffin wax, petroleum jelly, beeswaxes, and jojoba waxes, (4) components which build into the intercellular cement and seal the epidermis and restore the skin barrier, e.g., ceramides, cholesterol, lecithins, squalene, and fatty acids (143, 158, 242). Regular use of emollients ensures skin softening, and hydration and reduces the risk of skin-related issues (143). *Aloe vera* is widely used in traditional medicine as a moisturiser and anti-inflammatory, to treat wounds and skin inflammation (243). Due to bioactive compounds, such as amino acids (isoleucine, leucine) and saponin glycosides, have a gentle cleansing ability, proper for dry and itchy diabetic skin (244). Research proves, that daily care based on gentle cleansing and moisturising agents alleviates the symptoms of pruritus, erythema, cracking, and lichenification (158). Anti-itch therapies result mainly from a combination of ingredients with anti-inflammatory properties and those that normalise epidermal keratinisation. Anti-inflammatory effects are demonstrated by panthenol, valerian, coltsfoot, plantain, flaxseed, common chamomile, green tea, *Asiatic anthrax*, and resilience (242). Urea (above 10%), alpha-hydroxy acids—lactic and mandelic, and polyhydroxy acids with slight irritant potential have gentle keratoregulatory properties, indicated in skin care for DM (242).

An appropriate treatment plan, including correct application and dosing of moisturiser, anti-inflammatory, and keratoregulatory components, is essential to ensuring skin health and reducing the morbidity associated with DM skin conditions (143). The basic skin care procedure is based on the performance of a gentle enzymatic or low-concentration acid peel (5–15%) and the application of a moisturising mask in the form of alginate or collagen sheets. The treatment may be supplemented with the introduction of concentrated moisturising ingredients through a manual massage using occlusive oils (245, 246). Patients with controlled diabetes can benefit from physical methods that support the transport of active substances deep into the skin, such as iontophoresis based on direct current, sonophoresis using ultrasound, or needle-free mesotherapy. The choice of treatment depends on the specific type and severity of skin complications (242).

It is worth mentioning that maintaining proper skin hydration, along with a sufficient skin barrier against external irritants, increases the resistance of skin to cracking and infection (143). The guidelines highlight the use of suitable moisturisers, especially in foot care, because of the higher risk of medical complications (126, 143). In foot self-care, patients need to adhere to proper nail cutting and gentle callus removal to reduce the risk of wounds (126). Bacterial and fungal infections are common in DM. To avoid serious skin infections, all wounds or scratches need to be treated immediately with topical antibiotics and antifungal medications (247). In daily care, it is necessary to carefully dry areas prone to excessive moisture, such as the skin between toes or armpits. A wet environment promotes the development of infections (126, 143).

Natural products (NPs) have often served as a rich and promising reservoir of bioactive compounds for dermatological applications. NPs are defined as a natural compounds or substances produced by a living organism, including plants, animals, or fungi (248). They offer a unique opportunity not only to discover novel individual molecules but also to study the synergistic effects arising from the complex interplay of multiple substances within a single extract. This potential, combined with a growing consumer demand for sustainable and “green” products (230, 248), could position NPs as an attractive alternative in skin care. However, the path from a raw natural ingredient to a clinically approved treatment is fraught with challenges. The question of cost, for example, is highly dependent on context. While some natural sources may be inexpensive, the final product is not always cheaper than a synthetic equivalent (249). Factors such as certified organic sourcing, complex extraction processes, and the lack of reimbursement from insurance and healthcare systems can make them a more expensive option for consumers (250). The most significant barrier to the widespread medical adoption of NPs is the difficulty in performing robust clinical validation (250). Modern clinical trials are designed to test the safety and efficacy of standardised, single-molecule drugs. Natural extracts, by their very nature, are complex mixtures of numerous active molecules and their sub-products (248). This inherent complexity makes standardisation a formidable challenge, as the chemical profile can vary based on genetics, growing conditions, and processing methods. Consequently, designing rigorous, repeatable clinical trials that can definitively prove efficacy according to modern medical standards is exceptionally difficult, limiting their transition from cosmetic or traditional use to evidence-based therapeutic agents (250). Aside from these questions, some research has pointed to the antimicrobial and anti-inflammatory properties of natural botanical products and their ability to accelerate wound healing against various skin disorders (249). One of the reasons for skin lesions in DM is the overproduction of inflammatory mediators, such as TNF-*α*, IL-1*β*, and IL-6 (20, 33). Many natural products reduce inflammatory damage in skin tissue, leading to improved skin condition (251). Mangiferin (C-2 β-D-glukopyranozyl-1, 3, 6, 7-tetrahydroxyxanthone), lutein (β,*ε*-carotene-3,3′-diol), and curcumin (1,7-bis(4-hydroksy-3-metoksyfenylo)-1,6-heptadien-3,5-dion) have anti-inflammatory effects due to their antioxidant activity (20, 251–253). Studies conducted on mangiferin, a compound obtained from mango, indicate its regenerative and anti-inflammatory properties in skin inflammation diseases (20, 253). Mangiferin administration inhibits the inflammatory activity of macrophages and reduces oxidative damage in skin tissue, thus reducing dermatitis. A study on diabetic rats shows that mangiferin maintains tissue proliferation and growth, which is helpful in wound healing (20). The anti-inflammatory and antioxidant properties of mangiferin, when used in the formulation of a topical hydrogel delivery system, improve the regeneration of the skin layers (20). In another study, mangiferin applied in nanoemulsion reduced skin damage induced by 12-O-tetradecanoylphorbol-13-acetate (TPA). TPA is a protein kinase C activator that is applied topically to the skin and induces inflammation and epidermal hyperplasia (254). Thus, mangiferin improves skin inflammation and wound healing (255, 256). The dressing with carrageenan silver nanoparticles (CAgNPs) has also shown great properties in wound healing. CAgNPs acticoat stimulates epidermal reepithelialisation and has antibacterial properties against *Staphylococcus aureus* and *Escherichia coli* (19, 257). Lutein is present in dark and leafy green vegetables, like spinach, peas, lettuce, and broccoli. Mouse models have confirmed lutein’s properties in combating skin inflammation, including skin erythema and psoriasis (20). Curcumin is another natural product helpful in dermatitis (252). It originates from turmeric and is useful in combating skin lesions caused by oxidative damage and inflammation (20). Reducing lipid peroxidation and ROS production influences the anti-inflammatory effects of curcumin. Curcumin stimulates fibroblast migration and collagen synthesis and activates the production of growth factors and ECM proteins that promote wound repair in DM patients (258). Animal studies confirm that the application of curcumin nanofibres to a hard-to-heal wound accelerates its regeneration (20). In some reports, curcumin and resveratrol (most abundant in grape skin), with their anti-inflammatory, antimicrobial, and neuroprotective properties, are useful in alleviating cardiovascular disease or diabetes (20). Many recent studies confirm that compounds such as embelin (isolated from dried berries of Embelia ribes plants), naringenin (found in grapefruits, oranges, figs, or tomatoes), and quercetin (originating from grapes, apples, berries, onions, *ginkgo biloba*) have the potential for treating skin disorders (20, 259–261). Including these interventions in daily comprehensive skin care for individuals with DM ensures healthy skin and minimises the risk of complications. This will improve the comfort and overall quality of life in diabetic patients.

## 12 Conclusion

In this review, we have summarised the current knowledge on skin involvement in DM. The pathogenesis of cutaneous manifestations is multifactorial and results from biochemical, metabolic, vascular, and immune changes that occur in the diabetic state. The relationship between DM and skin disorders can be divided into: cutaneous diseases associated with diabetic angiopathy and neuropathy, manifestations strongly associated with DM, non-specific cutaneous symptoms, other skin disorders associated with DM, cutaneous infections in diabetic patients, and skin complications due to the therapy of DM (Table 1).

**Table 1:** Skin manifestations in DM.

Disease	Appearance	Pathogenic mechanisms	Prevalence	Location	Treatment
Diabetic angiopathy and neuropathy associated diseases					
Diabetic foot ulcer	Dry skin prone to cracks and fissures, impaired wound healing, and chronic ulcers (8, 11, 126, 128–130)	Angiopathy, ischemia, neuropathy, and skin infection	19–34% diabetics (8, 11, 126, 128–130)	Feet (8, 128–130)	Proper foot self-care, appropriate foot hygiene, proper footwear, calluses treatment (8, 11, 126, 128–130)
Diabetic gangrene	Necrosis from ulceration, moist, swollen, soft, rotten, and dark tissue (128, 129, 137, 140)			Feet (128, 129, 137, 140)	Improved glycaemic control, antibiotics, surgical interventions, autoamputation (128, 129, 137, 140)
Diabetic dermopathy	Oval, dull, red papules, atrophic, hyperpigmented patches, and plaques with a fine scale (8)		50% diabetics (142)	Pretibial area, thighs (8, 142, 143)	Self-resolving (142, 143)
Manifestations strongly associated with DM					
Yellow palms and soles	Yellowing of the skin (12, 146, 147)	Impaired *β*-carotene conversion	40–50% diabetics (12, 146, 147)	Palms and Soles (12, 146, 147)	Improved glycaemic control (12, 146, 147)
Acanthosis nigricans	Dark-brown plaques, lichenified, velvety, raised from the skin (8, 11, 142, 148)	Hyperkeratinisation and melanin overproduction	50% diabetics (142)	Axilla, neck, and groin (8, 11)	Improved glycaemic control, keratolytic agents—isotretinoin, salicylic acid, retinoids, urea (11, 142)
Bullosis diabeticorum	Tense, non-inflammatory vesicles and bullae (12, 142, 143, 146)	Vascular complications	0.5% diabetics (8, 142, 144)	Hands and feet (8, 142)	Self-resolving (8, 142)
Diabetic thick skin	Grouped, small, indurated papules, with reduced joint mobility (8, 146)	Collagen disorders	50% diabetics (146)	Extensor surface of the fingers, knuckles, periungual surface (12, 146)	Improved glycaemic control, no specific therapy (12, 146)
Scleredema diabeticorum	Painless, symmetrical and diffuse thickening of the skin, with reduced joint mobility (12, 142, 146)	Collagen disorders	2.5% diabetics (8, 146)	Face, trunk, neck, and upper limbs (8, 12)	Improved glycaemic control, oral glucocorticoids, pentoxifylline, prostaglandin E1, methotrexate (12, 142, 146)
Necrobiosis lipoidica	Erythematous papules, a well-demarcated plaque with an atrophic centre (8, 12)	Microvascular ischemia and collagen disorders	0.3%–1.2% diabetics (8, 11, 12)	Shins (11, 146)	Self-resolving (11, 12, 142, 146)
Non-specific symptoms associated with DM					
Acrochordons	Pedunculated, hyperpigmented lumps (8, 11, 148)	Increased proliferation of keratinocytes	23% diabetics (142)	Neck, armpits, periorbital area (8)	Excision, electrotherapy, or cryotherapy (11, 12)
Rubeosis faciei diabeticorum	Erythema, vascular oedema, telangiectasias (11, 153)	Microangiopathy	59% diabetics (153)	Face and neck (11)	Improved glycaemic control (8, 11)
Eruptive xanthomas	Multiple reddish-yellow dome-shaped papules with a tendency to sudden eruption (8, 11)	Hypertriglyceridemia	No data	Extremities, buttock region, and hands (8, 11)	Improved glycaemic and lipid control, laser therapy, cryosurgery, or surgical excision (8, 11)
Acquired reactive perforating collagenosis	Pruritus, erythematous papules, and hyperkeratotic plaques with a centralised keratin plug (12, 79, 155)	Collagen fibre degeneration	No data	Arms and legs (12, 79, 155)	Topical and oral retinoids, allopurinol (12, 79, 155)
Keratosis pilaris	Pink-red, monomorphic, follicular papules (12, 142, 158)	Increased proliferation of hair follicle keratinocytes	No data	Upper arms, thighs, face, back, and buttocks (12, 142, 158)	Topical exfoliators, emollients, laser therapy (12, 142, 158)
Pruritus	Dry skin (xerosis) (11, 12, 158)	Polyneuropathy	No data	Generalised (11, 12, 158)	Emollients, topical corticosteroids, antihistamines (11, 12, 158)
Other disorders associated with DM					
Vitiligo	Skin discolouration (12, 146, 159)	Autoimmune and neurohormonal factors	1–7% diabetics (146, 159)	Lower limbs, face, neck, and trunk (12, 146)	Topical corticosteroids, treatment with ultraviolet B light (11, 12, 146, 158, 159)
Granuloma Annulare	Multiple, pink-red papules up to 5 cm in size, of arciform and annular shape, with central, non-atrophic clearing (8, 12, 142, 146)	Collagen fibre degeneration	10–15% diabetics (12, 146)	Joints and dorsal hands and feet (8, 12)	Corticosteroids, PUVA therapy, or cryotherapy (8, 12, 142, 146)
Lichen Planus	Firm, erythematous, polygonal, pruritic papules with shiny, whitish streaks on the surface (11, 12)	Autoimmune basis	25% diabetics (11, 12)	Wrists and ankles (11, 12)	Corticosteroids, calcineurin inhibitors, phototherapy, systemic retinoids (11, 12)
Bacterial infections associated with DM					
Folliculitis	Tender, red spot, with a surface purulent pustule in hair follicles (12, 143)	Increased skin surface pH, bacterial infiltration, and induced inflammation	20–50% of diabetics will develop some cutaneous infections, but the most common bacterial infection is DFI, occurring in 4% of diabetics (11, 12, 52)	On hair-covered skin (12, 143)	Topical antibiotics (12, 143)
Abscesses	Painful, red, swollen purulent bumps—boils (12, 143)			Face, neck, armpits, buttocks, and thighs (12, 143)	Surgical treatment, antibiotics (12, 143)
Impetigo contagiosa	Honey-coloured crusts, and epidermal erosion (12)			Face and extremities (12)	Antibiotics (12)
Ecthyma	Small, brown-black, crusted sores with surrounding erythema, rapidly progress (12, 167)			Lower legs or feet (12, 167)	Antibiotics, local antiseptics (12, 167)
Cellulitis	Warm tenderness, brilliant erythema, fever (12, 168)			Generalised (12, 168)	Antibiotics (12, 168)
Necrotising fasciitis	Early erythema progresses to a severe painful haemorrhagic blister (12, 169)			Lower extremities (12, 169)	Surgical treatment, antibiotics (12, 169)
Erythrasma	Well-demarcated, erythematous lesions that may turn brownish, with central clearing and raised edges in skin folds (8, 171)			Groin folds, axillae, and gluteal cleft (8, 171)	Antibiotics (8, 171)
Diabetic foot infection	Wound/ulcer with pus, redness, swelling, pain, or warmth (12, 52, 172, 173)			Feet (12)	Antibiotics, wound debridement, amputation (12, 52, 172, 173)
Fungal infections associated with DM					
Candidiasis	Pruritic erythematous rash, vesicular-pustular lesions, perforation, and fissures (12, 143)	Increased skin surface pH, fungal colonisation	20–50% of diabetics will develop some cutaneous infections, but the most common fungal infections are candidiasis (8, 11, 12)	Interdigital areas, nails, mucosa (12, 143)	Antifungal medications (12, 143)
Dermatophytosis	Erythematous, horny or bullous lesions with itching or pain (8, 12)			Generalised (8, 12)	Antifungal medications (8, 12)
Onychomycosis	White papules and plaques, and erythematous erosions (8, 12)			Nails (8, 12)	Antifungal medications (8, 12)
Mucormycosis	Sinusitis with purulent nasal discharge, rash, facial erythema, oedema, and cellulitis with systemic fever (8, 12, 182)			Face (8, 12, 182)	Surgical intervention, amphotericin B (8, 12, 182)
Cutaneous reactions to insulin					
Lipoatrophy	Loss of local subcutaneous fat—small dent at the injection site (12, 188)	Inflammatory factors	10–55% diabetics (12, 188)	Site of insulin injection (12, 188)	Discontinuing injections at this site, corticosteroids, betamethasone (12, 188)
Lipohypertrophy and subcutaneous nodules	Increase of local subcutaneous fat-soft nodules of variable size (12)	Insulin-dependent activation of adipocytes	27% diabetics (12)	Site of insulin injection (12)	Discontinuing injections at this site (12)
**Insulin allergy**	Pruritic urticarial papules, subcutaneous inflammatory nodules, with temporary itching or pain, rare life-threatening anaphylaxis, and angioedema (194, 195, 197)	Immune basis	0.1–3% of diabetics (194, 195, 197)	Cutaneous reactions at the site of insulin injection, and generalised reactions (194, 195, 197)	Antihistamines, leukotriene inhibitors, and topical steroids (194, 195, 197)
Cutaneous reactions to oral antidiabetic agents					
Psoriatic eruptions	Scaly, erythematous plaques, sharply demarcated (187)	Immune-mediated mechanism	No data	Extensor surfaces (187)	Avoiding or regulating the dose of the drug (187)
Leukocytoclastic vasculitis	Haemorrhagic lesions, both papules and bullae (187, 200)	Inflammatory condition		Generalised (11, 187, 200)	
Photosensitivity	Excessive sunburn reactions with erythema, itching, and burning sensation (199, 202)	Immune-mediated mechanism		Generalised (199, 202)	
Pemphigus vulgaris	Blisters (203, 204)	Immune-mediated mechanism		Cutaneous and mucosal (203, 204)	

DFI, diabetic foot infection.

Chronic uncontrolled hyperglycaemia contributes to epidermal barrier abnormalities, reduced stratum corneum hydration, increased skin pH, and altered keratinocyte and fibroblast activity, resulting in impaired wound healing and secondary infections. All these conditions promote cutaneous inflammation and reduce the comfort of life. Maintaining skin integrity and preventing skin complications are the gold standard in diabetic skin therapy. It is important to provide optimal care for the diabetic patient to increase their quality of life and prevent severe complications.

There is still a lack of biomarkers for identifying skin DM complications. The use of omics technologies may allow for the discovery of new therapeutic targets, thereby enabling earlier diagnosis and risk stratification. Longitudinal studies on skin biomarkers in DM are essential.
